# Developing a brief motivational intervention for young adults admitted with alcohol intoxication in the emergency department – Results from an iterative qualitative design

**DOI:** 10.1371/journal.pone.0246652

**Published:** 2021-02-08

**Authors:** Jacques Gaume, Véronique S. Grazioli, Sophie Paroz, Cristiana Fortini, Nicolas Bertholet, Jean-Bernard Daeppen

**Affiliations:** 1 Department of Psychiatry, Addiction Medicine, Lausanne University Hospital, Lausanne, Switzerland; 2 Department of Vulnerabilities and Social Medicine, University Center for General Medicine and Public Health, Lausanne, Switzerland; The University of Sydney Faculty of Medicine and Health, AUSTRALIA

## Abstract

**Background:**

Unhealthy alcohol use among young adults is a major public health concern. Brief motivational interventions for young adults in the Emergency Department (ED) have shown promising but inconsistent results.

**Methods:**

Based on the literature on brief intervention and motivational interviewing efficacy and active ingredients, we developed a new motivational intervention model for young adults admitted in the ED with alcohol intoxication. Using an iterative qualitative design, we first pre-tested this model by conducting 4 experimental sessions and 8 related semi-structured interviews to evaluate clinicians’ and patients’ perceptions of the intervention’s acceptability and feasibility. We then conducted a consultation meeting with 9 international experts using a nominal group technique. The intervention model was adjusted and finally re-tested by conducting 6 new experimental sessions and 12 related semi-structured interviews. At each round, data collected were analyzed and discussed, and the intervention model updated accordingly.

**Results:**

Based on the literature, we found 6 axes for developing a new model: High level of relational factors (e.g. empathy, alliance, avoidance of confrontation); Personalized feedback; Enhance discrepancy; Evoke change talk while softening sustain talk, strengthen ability and commitment to change; Completion of a change plan; Devote more time: longer sessions and follow-up options (face-to-face, telephone, or electronic boosters; referral to treatment). A qualitative analysis of the semi-structured interviews gave important insights regarding acceptability and feasibility of the model. Adjustments were made around which information to provide and how, as well as on how to deepen discussion about change with patients having low levels of self-exploration. The experts’ consultation addressed numerous points, such as information and advice giving, and booster interventions.

**Discussion:**

This iterative, multi-component design resulted in the development of an intervention model embedded in recent research findings and theory advances, as well as feasible in a complex environment. The next step is a randomized controlled trial testing the efficacy of this model.

## Introduction

Alcohol use is the first cause of mortality among adolescents and young adults in the world, and is related to 20–25% of all deaths among this age group in Europe [1]. Heavy episodic drinking (HED, i.e. drinking 6 standard drinks or more [>60 grams of pure alcohol] on a single occasion) and acute alcohol intoxication are associated with an increased risk of injuries, trauma, violence, risky sexual behaviors, and other negative health outcomes, especially among young adults [2]. HED during adolescence has also been related to an increased risk of alcohol dependence, other substance abuse, psychiatric comorbidities, and social difficulties in adulthood [3].

Emergency Department (ED) admissions related to alcohol intoxication represent a large burden on the ED clinical teams and account for a significant part of the resources in EDs [4–6]. In Switzerland, ED admissions for alcohol intoxication have increased over the last decade, among all age groups (+11%), but particularly among adolescents and young adults (+57% among 10–23 years olds; [7]). At the Lausanne University Hospital ED, the number of young adults between 18 and 30 years old admitted with a positive blood alcohol concentration (blood alcohol concentration ≥ 0.5 gram/liter) increased fourfold between 2000 and 2011 [4]. Moreover, recent results of a study on young adults admitted in this ED with alcohol intoxication showed that over the next 7 years, about a half of them were re-admitted in this ED (1/4 for a new alcohol intoxication episode), 36.8% were unemployed, 56.9% reported hazardous alcohol use, 15.1% alcohol dependence, 18.6% depression, 15.4% anxiety disorder, 80.2% smoked tobacco during the last year, 53.1% used cannabis during the last year, and 22.6% used cocaine during the last year [8].

While young adults incur significant harm due to unhealthy alcohol use, studies of the natural history of alcohol use disorders have shown that the likelihood of such disorders is lower among younger individuals than it is among older individuals; if present, they are probably milder in severity [e.g. 9]. Therefore, secondary prevention interventions are likely to be of substantial benefit with younger individuals [10]. Reviews on strategies targeting alcohol use show that brief interventions (BI) are among the few effective preventive strategies and the most cost-effective strategy among person-centered approaches [11, 12]. Structured brief advice is the most common BI and appears to lend itself to wide implementation, though it might not be adequate for addressing more severe alcohol problems [13]. The other principal type of BI is brief motivational intervention (BMI), i.e. brief adaptations of motivational interviewing (MI). MI combines the person-centered counseling approach originally developed by Carl Rogers with a behavioral focus on resolving ambivalence in the direction of change [14]. McCambridge and Rollnick [13] have proposed that targeting alcohol problems directly with high quality MI as a BI is a promising route for further study. MI is an evidence-based treatment for adult alcohol problems, demonstrating equivalence in effectiveness to more intensive psychological treatments while showing greater cost effectiveness [e.g. 15, 16]. Adolescents and young adults are particularly receptive to motivational methods because they include acceptance, avoidance of argumentation and hostile confrontation, and eschew giving lectures or ultimatums [17].

Two recent systematic reviews addressed the efficacy of BMI conducted in the ED for young adults and both found mixed findings [18, 19]. Newton, Dong [19] also noted poor study quality precluding firm conclusions for many comparisons. A recent meta-analysis of alcohol BMIs for adolescents and young adults [20], however, showed that BMI led to significant reductions in alcohol consumption (effect size = 0.17) among young adults 19 to 30 years old. Tests of intervention characteristics as potential moderators showed smaller but significant effect size in the ED settings (effect size = 0.11).

The studies summarized above typically used a systematic screening process to include participants. Therefore, results might not be generalizable to populations admitted in the ED while intoxicated. One recent systematic review [21] investigated the efficacy of interventions among this specific population. The authors found 8 studies, including 4 studies comparing MI to standard care among young adults. Three of these showed results favoring MI. Monti and colleagues [22] found significant differences on alcohol-related re-injuries and problems, and drinking and driving among 94 young adults (aged 18–19). Smith and colleagues [23] found significant differences on alcohol use and on alcohol-related problems among 151 young adults with facial injury (aged 16–35). Spirito and colleagues [24] found no significant effects among 152 adolescents (aged 13–17) overall, but found significant effects on alcohol drinking days and binge drinking when limiting analyses to those reporting pre-existing problematic alcohol use. Only one study had null findings [25]; it included an older sample (18 to 45) comprised of motor vehicle crash victims, excluded patients with higher alcohol problem severity, and evaluated a 15–20 minute MI as a complement to a 5–25 minute health interview. Wicki and colleagues [21] concluded that MI had a clear added value when compared to standard care, at least at short-term follow-up. However, they noted that it remained unclear which elements were related to efficacy.

If current findings have shown mixed effects for BIs among young adults in the ED, and promising effects for MI among those intoxicated, more advanced research is needed on how to optimize this secondary prevention opportunity. This includes information on key intervention components, ideal quality of intervention delivery, and which particular sub-groups are most likely to benefit from the intervention. We believe this is an important direction for intervention research with the potential to improve existing models [26].

This study thus aimed to develop a new motivational intervention model for young adults admitted in the ED with alcohol intoxication. The present article reports the first phase of a larger project using a general mixed methods approach [e.g. 27], in which we will later test the efficacy of this new model using a randomized controlled trial (registered as http://www.isrctn.com/ISRCTN13832949) and evaluate the mechanisms of the intervention effects. This first phase consisted of an iterative process to develop and pre-test the intervention model through qualitative evaluation and refinement of the intervention tool.

## Materials and methods

**Fig 1:**
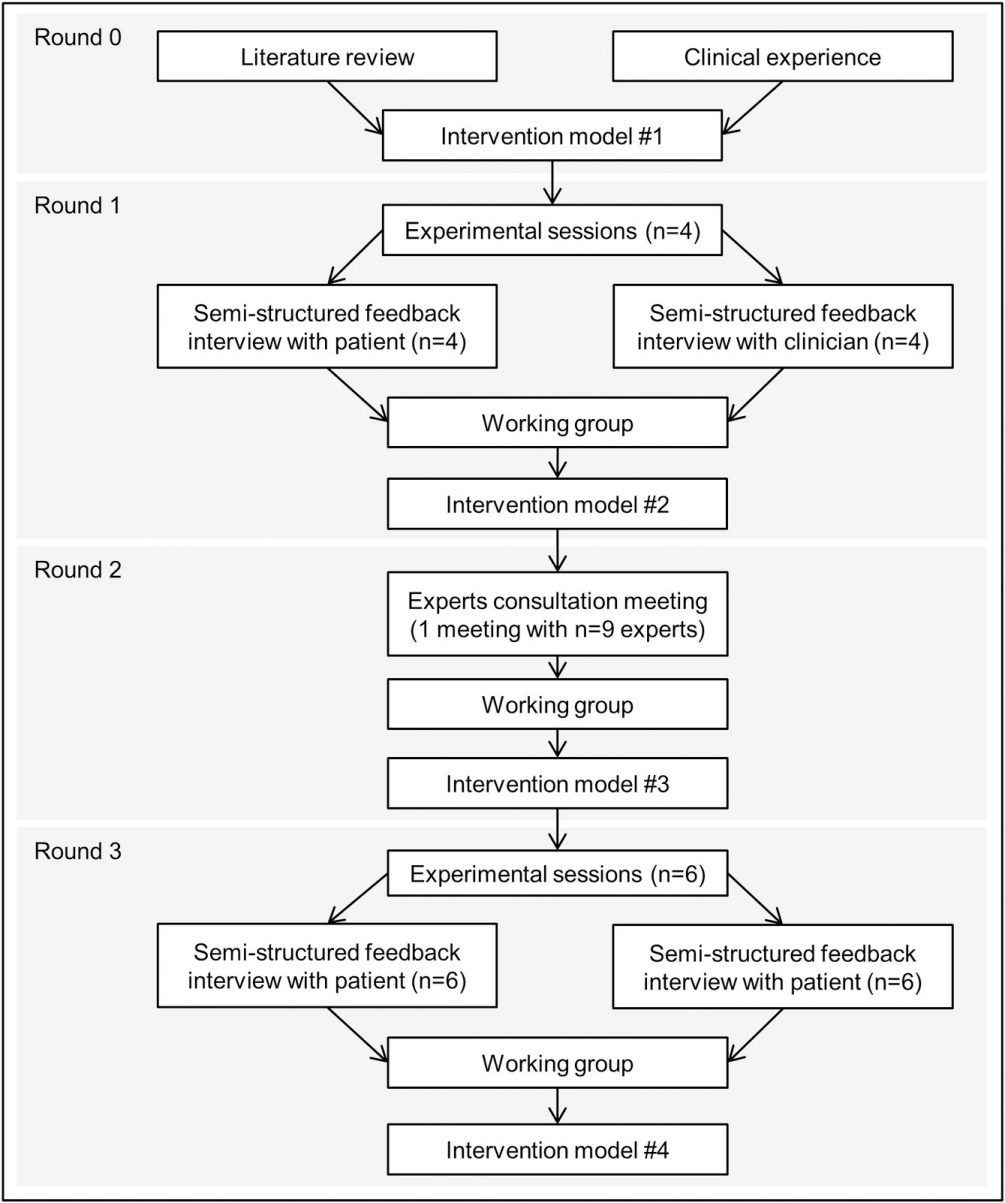
Design of the brief motivational intervention development process.

Our iterative process comprised four rounds (Fig 1): Round 0- first draft of the intervention model based on a literature review and clinical experiences; Round 1- experimental sessions testing the intervention model in the real world and collecting clinicians’ and patients’ feedback on their experience; Round 2- expert consultation; and Round 3- a second round of experimental sessions. At each round, data collected were analyzed and then discussed during working group meetings (the working group was composed of the authors of the present article). These meetings intended, at each round, to update the model of the intervention according to the round results.

### Round 0 –definition of intervention model #1

This preliminary round was conducted in 2015 and comprised a review of the literature and the setting-up of the first intervention model.

### Round 1 –experimental sessions

Round 1 aimed at testing the Intervention model 1 in the real world and collecting clinicians’ and patients’ feedback regarding the intervention. Four experimental sessions were conducted to examine acceptability and feasibility of delivery.

#### Participants and setting

Clinicians (*n* = 2) were psychologists who were trained to deliver the Intervention model 1 in a workshop involving role-plays with the members of the working group team. Patients (*n* = 4; 50% female, mean age = 22.5) were the first four patients corresponding to the study’s inclusion and exclusion criteria. A sample of 4 sessions and 8 semi-structured interviews with each patient and clinician involved was planned a priori, taking into account the exploratory nature of the study and its iterative process. Recruitment took place at the ED during six mornings between July 28 and August 28, 2016. Fourteen patients met inclusion criteria, i.e. aged 18–35, admitted to the ED for any cause, and with alcohol intoxication (> 0.5 gram/liter BAC). Of those, 7 were excluded (psychiatric contra-indication, *n* = 4; medico-legal admission, *n* = 1; currently in alcohol treatment, *n* = 1; not fluent in French, *n* = 1), 1 left the ED prior to receiving the information session and 2 refused to participate.

#### Data collection procedures

Eight individual semi-structured interviews were conducted. We interviewed both patients and clinicians on their experience and perceptions of the intervention using an interview grid (S1 File). The interview with each patient was conducted directly after the BMI; it was immediately followed by the interview with the clinician who had provided the intervention. Interviews lasted between 20 and 30 minutes each. All interviews were conducted by the second author. We audio-recorded and transcribed verbatim interviews and removed all names and identifying information to guarantee confidentiality. All procedures were approved by the local Ethics Committee (*Commission cantonale d’éthique de la recherche sur l’être humain*–CER-VD, Protocole 2016–01476) and all participants provided written informed consent.

#### Qualitative data analysis plan

Qualitative data were subject to conventional content analysis using ATLAS.ti 7 qualitative data analysis program [28]. Conventional content analysis enables description of qualitative data through a systematic process of coding and classification [29]. Qualitative data were reviewed by the second author to identify recurring categories. According to procedures proposed by Charmaz [30], the initial coding was conducted using a line-by-line technique aiming to narrate the actions occurring in the interviews. After the initial coding, a codebook was created, wherein incident-by-incident codes were pooled, and idiosyncratic or redundant codes were collapsed or eliminated. After the codebook was created, the second author rated all qualitative data again. We present quotes for illustration of categories emerging from the analysis. We translated selected quotes from French into English; original quotes are presented in S1 Table (for reference, quotes are numbered Q1 to Q32).

### Round 2 –experts’ consultation

In this round, international experts in MI and BMI were consulted about the intervention model. To best capitalize on experts’ experience and insights, we used a consultation group meeting format in order to discuss the practicality, appropriateness, and theoretical soundness of the model, by generating propositions and working on a consensus on these for an amended model to be tested in Round 3.

Participants and setting. This group meeting took place on Thursday 22 September 2016, in Lausanne, Switzerland. We took advantage of the 13th Annual Conference of the International Network on Brief Interventions for Alcohol Problems (INEBRIA), which was hosted in Lausanne at that moment. Among the 11 experts invited to participate, 2 were interested in participating but were not available to attend, and 9 accepted invitation and participated. These were (in alphabetic order): Prof. Gail D’Onofrio, MD, MS, Professor of Emergency Medicine and Chair of the Department of Emergency Medicine, Yale University, USA; Prof. Craig Fields, PhD, Associate Professor, Department of Psychology, University of Texas at El Paso, USA; Dr. Jennis Freyer-Adam, PhD, *Privatdozentin*, Institute of Social Medicine and Prevention, University of Greifswald, Germany; Prof. Nick Heather, PhD, Emeritus Professor of Alcohol and Other Drug Studies, Department of Psychology, Northumbria University, UK; Prof. Molly Magill, PhD, Assistant Professor of Behavioral and Social Sciences, Center for Alcohol and Addiction Studies, Brown University, USA; Prof. Jim McCambridge, PhD, Professor and Chair in Addictive Behaviours & Public Health, Department of Health Sciences, University of York, UK; Prof. Peter Monti, PhD, Professor of Behavioral and Social Sciences, Director of the Center for Alcohol and Addiction Studies, Brown University, USA; Prof. Stephen Rollnick, PhD, Honorary Distinguished Professor at Cochrane School of Primary Care & Public Health, Cardiff University, UK; Prof. Richard Saitz, MD, MS, Professor of Medicine and Epidemiology and Chair of the Department of Community Health Sciences, Boston University, USA.

#### Research approach and analysis

The meeting was a 4-hour workshop which was led using a participatory research approach inspired by the nominal group technique as a consensus method [31]. The fourth author (CF) facilitated the discussion, and the first (JG) and last author (JBD) attended the meeting to present data and models, and answer clinical and logistical questions. We first presented the meeting agenda, the study context, and the clinical setting. The intervention model was then presented to the group. We then used nominal group technique (NGT) to address 3 predetermined topics (see below). NGT [see e.g. 32] is a consensus method used for exploring expert views, problem-solving, idea-generation, or determining priorities. NGT involves four steps to address each predetermined topic: 1) *Generating ideas* (the facilitator presents the question/issue and each group member silently generates and writes down ideas); 2) *Recording ideas* (each group member exposes his/her idea(s), the facilitator records them on a screen); 3) *Discussing ideas* (each idea is discussed to determine clarity and importance); 4) *Voting on ideas* (each group member votes privately to prioritize ideas). For the voting phase, experts were asked to select the 5 most important propositions, and then to rank them from the most to the least important on a scale from 1 (least important) to 5 (most important). Votes were collected and scores for each proposition were summed across experts. Propositions with higher scores were considered the most favored group actions or ideas in response to the question posed.

### Round 3 –experimental sessions

Round 3 replicated methods used in Round 1 but by testing our new intervention model (i.e. model #2, see Fig 1) with new patients.

#### Participants and setting

Clinicians (*n* = 2) were the same psychologists as in Round 1, trained in using the new features of the intervention model. Patients (*n* = 6; 33% female, mean age = 22.3) were the first six patients corresponding to study inclusion and exclusion criteria. Again, this sample of 6 sessions and 12 semi-structured interviews was planned a priori, taking into account the exploratory nature of the study and its iterative process. The full process resulted in 20 semi-structured interviews, which effectively led to data saturation. Recruitment took place at the ED during 10 mornings between October 8 and November 11, 2016. Twenty-one patients met inclusion criteria. Of those, 10 were excluded (psychiatric contra-indication, *n* = 6; currently in alcohol treatment, *n* = 3; not fluent in French, *n* = 1), 3 refused to participate, 1 left the ED prior to receiving the information session and 1 was not approached by the research team by lack of time.

#### Qualitative data analysis plan

Similar to Round 1, qualitative data were subject to content analysis. The second author first reviewed a subset of interviews (2 with the clinicians and 2 with the participants) to identify new categories that did not appear to fit into the original codebook. The codebook was modified accordingly. Then, the second author rated all qualitative data from Round 3.

## Results

### Round 0 –definition of intervention model #1

This preliminary round aimed at drafting the first model of the intervention. This model was based on a recently published literature review on BI mechanisms [33] and MI mechanisms [34], on wider addiction and psychotherapy process research (see references below), and on the clinical experience of our own staff involved in ED alcohol liaison. In particular, 6 components were selected. These components were integrated in an intervention manual for implementation and testing in Round 1. We summarize these 6 components below.

#### Relational factors: Empathy, acceptance, collaboration, and avoidance of confrontation

Relational factors can be significant determinants of addiction treatment outcome [35]. Empathy has been relatively well established as an active ingredient in the general psychotherapy literature [e.g. 36] and in addiction treatment [37]. Several interpersonal skills (e.g. acceptance, empathy, collaboration and support of client autonomy) have been related to client involvement in MI [38–40] and to alcohol outcomes in BMI [41–43]. Reflective listening is an important technique to deepen understanding of the patient’s perspective [14]. It has been related to more discussion on change (change talk) and enhanced outcomes [43–45]. On the other hand, confrontation has been found to be particularly unhealthy, in that it decreases client change talk, reinforces resistance and sustain talk, and in some studies directly affects client outcomes negatively [43, 44, 46, 47].

#### Personalized feedback

Early BI models have focused explicitly on feedback on risk or harm as a tool for instigating change [48]. Meta-analytic findings are supportive of the use of feedback [48–50]: the interventions that include feedback have significantly better outcomes than those who do not. Studies that have experimentally investigated this question produced more mixed, but promising findings [51–54]. In a fundamental study of MI, providing feedback in a non-confrontational MI style doubled client change talk and halved resistance [47].

#### Enhance discrepancy

To develop discrepancy between the individual’s current behavior and their broader life goals and values is a core feature of MI [14]. In one empirical study [55], discrepancy measures were significantly increased following BMI and were correlated with alcohol outcomes among heavy-drinking college students. In an ED-based alcohol BI study, Walton, Goldstein [56] showed that attributing injury to alcohol consumption moderated the intervention effect, suggesting that highlighting the connection between alcohol and injury can increase the effectiveness of the intervention. By extension, evocation of the current situation (alcohol intoxication, potentially alcohol-related injury) in contrast with broader life goals and values might be an important mechanism of change.

#### Evoke change talk/strengthen ability and commitment to change

MI has been described as a collaborative conversation style for strengthening a person’s own motivation and commitment to change [57], and central to it is the hypothesis that people are more likely to be persuaded by what they hear themselves say [14, 58]. Empirical support for change talk evocation as an active ingredient in MI has been accumulating [44, 59, 60]. Among the different dimensions of change talk, ability to change has been linked to confidence to change and self-efficacy, a central principle in MI [14]; this dimension has been shown to predict enhanced outcomes in ED patients [41] and young adults [61, 62].

#### Change plan completion

Completion of a plan to change alcohol use is an MI component resulting in verbal statements of intention, and a written contract for behavior change [63]. Magill and colleagues [63] showed that change plan completion was related to higher therapist MI skills and client change talk within the session. Lee and colleagues [64] showed that good-quality change plan were associated with better outcomes, regardless of pre-intervention readiness to change.

#### More time: Longer sessions and/or booster sessions

Systematic reviews on alcohol BI and BMI have not clearly determined the optimal intervention length [65], even if more intensive interventions tended to yield overall more favorable results in ED-based interventions [66]. One study recently investigated the efficacy of 3 strategies of gradual intensity to address heavy drinking among injured patients [67]. Findings showed that BMI plus telephone booster showed significant reductions in alcohol use and binge drinking compared with brief advice or BMI alone. This evidence suggests that longer interventions including booster sessions are more effective in an ED setting.

### Round 1 –experimental sessions

Categories yielded in the current analysis reflected participants’ and clinician’s perceptions of the intervention’s feasibility, acceptability and utility.

#### Participants and clinicians’ perceptions of the intervention feasibility

*Suitability of the intervention in the ED*. Participants and clinicians uniformly reported that the ED was overall suitable for the intervention. They noted both disadvantages and advantages related to this setting. On the one hand, clinicians reported that it was feasible to provide the intervention in this setting, even though it required adapting to specificities related to the ED, such as noise or being interrupted several times by other staff or participants’ relatives during the intervention. One clinician explained, “*It can be difficult to get back on track*, *to know where we were*, *what I had in mind that I wanted to say*, *what the patient was saying*” (Q1). Participants’ tiredness was mentioned as another disadvantage by clinicians who sometimes felt urged to end the intervention. Relatedly, a participant mentioned her “*feeling that by giving [us] time*, *[she] was wasting time to able to get out [of the emergency department] sooner*” (Q2). Tiredness was also frequently mentioned as a barrier to participate in the intervention; one participant explained for instance that although he found the intervention interesting, he first did not want to participate because he felt too tired. That said, one participant noted that the setting (i.e., ED) eased the discussion that might otherwise be uncomfortable. Another participant corroborated this point of view noting, *“I cannot see another context in which such a discussion could have happened*” (Q3). On a similar note, the context was perceived as a change trigger by this patient: “*It is not*, *well*, *it is really not trivial and I think one should be a bit I don’t know*, *simple-minded [laughing]*, *stupid to not*, *well*, *not seeing this like*, *[…] it is not anything*, *it is*, *I am at the emergency department*, *it is not for nothing and there is a problem*, *here*, *it really makes you think*, *operate a change*” (Q4).

**Fig 2:**
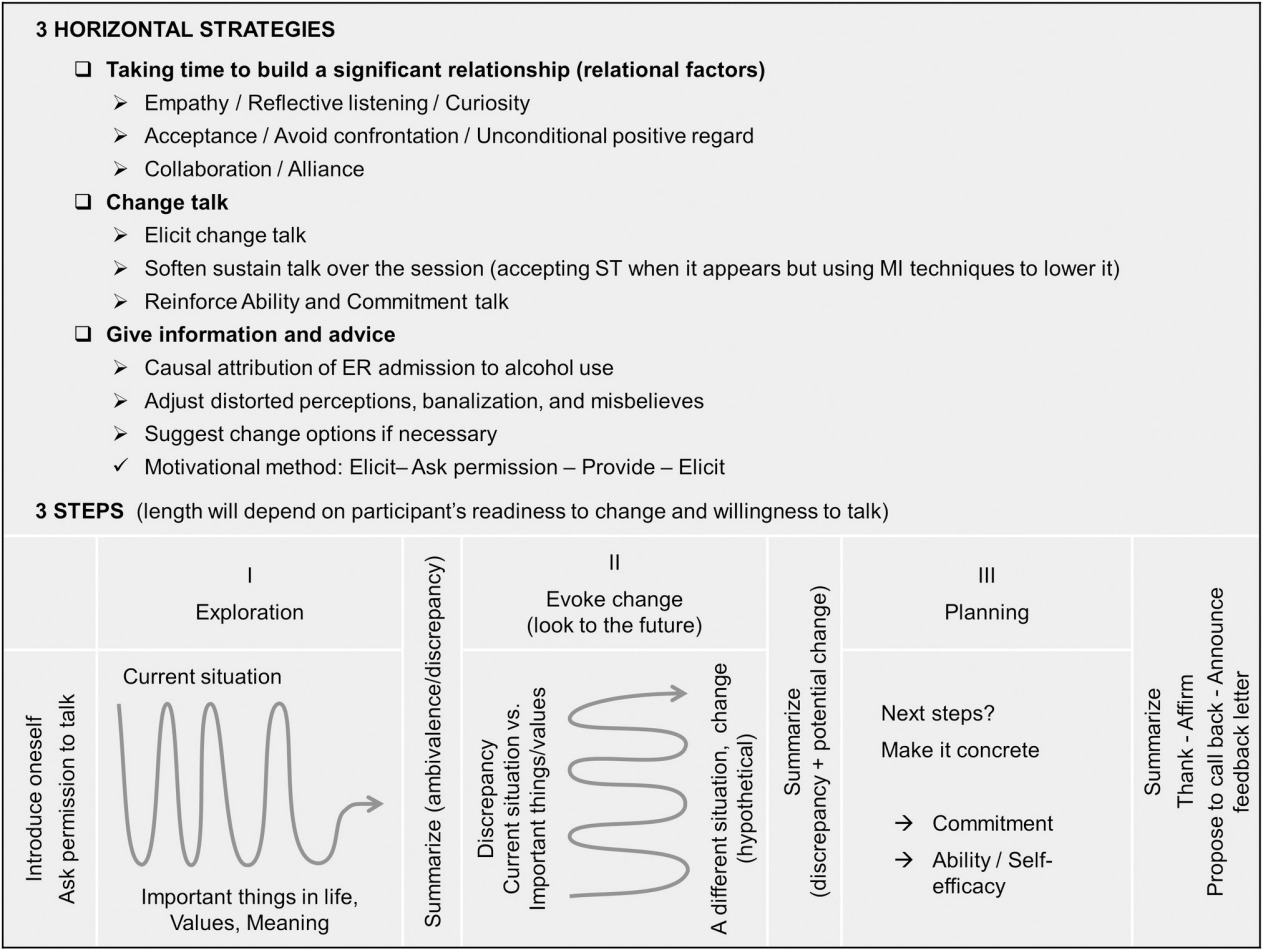
Schematic outline of the definitive intervention model.

*Perceptions of the intervention model applicability*. Both clinicians indicated that the model was overall applicable in the ED setting. They described the model as “*flexible*” and “*tailored to the situations*” they had met. They also mentioned that the three steps (Fig 2) made sense and were applicable, although they did not systematically apply them chronologically: “*Let’s say that I had this [the three-step aspect of the model] in mind*, *then it depends upon what the person brings that we go back and forth*, *but otherwise it seems coherent enough*, *a logical construction*” (Q5). Notably, both clinicians shared difficulties in deepening discussion at some point during the interviews. A clinician mentioned for instance that she “*didn’t have the impression of being able to go very far in the discussion about alcohol*, *about what brought [the patient] here at the emergency department that day*” (Q6). More generally, addressing plan to change patterns of alcohol use was uniformly reported as challenging by clinicians: “*When I asked her how she saw her drinking in the future*, *she couldn’t really answer*, *and then*, *well she said that it would get better and better*, *but then in the planning phase there wasn’t really a concrete objective*, *well*, *but… I asked anyway how important it was for this person*, *how much she felt confident*, *so*, *well*, *I asked*, *it was ok*, *but it’s true that there wasn’t necessarily something concrete either”* (Q7).

#### Participants’ and clinician’s perceptions of the intervention’s acceptability

*Participants’ general acceptability of the intervention*. Overall, the intervention was well received among participants, who reported liking it. They all mentioned feeling at ease and experiencing the intervention well. Although most participants noted that the intervention length was adequate, one participant considered that it was too long and that it may represent a barrier to its acceptability: “*In my opinion*, *this could be very dissuasive for some people*. *When you see the 20 to 60 minutes range*, *it’s quite scary*!” (Q8). Echoing this point of view, another participant noted, “*I am sure there are people who actually want to do your thing [the intervention] but who tell themselves no*, *they will waste their time*, *that it will make them waste even more time*” (Q9).

*Participants’ perceptions of the clinicians*. Most participants perceived the clinicians positively. Clinicians were commonly described as “*very kind*,” “*open*,” “*soothing*”, and “*understanding*.” Participants frequently reported feeling at ease and understood, and appreciated the discussion they had with the clinicians: “*It was not*, *how can I say*, *intrusive*, *or*, *she was not looking for something*, *she was not trying to get something and*, *well it was a relationship […] I could really talk normally*” (Q10). Other positive perceptions mentioned by some participants included professionalism and attentiveness. Finally, one participant considered that the clinician was too neutral and noted, “*She [the clinician] doesn’t judge*, *she just takes information*” (Q11). This participant disclosed, “*Yeah*, *I would have been interested in having her point of view regarding what I said; because at the end*, *she just listened to me*, *she asked me questions*, *but she gave me no opinion actually*” (Q12).

*Participants and clinicians’ perceptions of the intervention’s content*. Overall, participants held positive perceptions of the intervention’s content. The intervention was described as “*well-crafted*” and “*complete*.” However, perceptions regarding alcohol-related information content were more diverse. Although alcohol-related information (e.g., personalized alcohol-related feedback, harm-reduction tips) was not systematically provided in this first round of experimentation, participants and clinicians were asked about this content’s fit and acceptability. Half of the participants considered that providing alcohol-related information might be useful. For instance, a participant explained, “*It [a personalized alcohol-related feedback] may be useful*, *yes […] to indicate*, *well*, *to know where we really stand*, *because*, *I think that many people believe they do not drink much but at the end they’re already good drinkers*” (Q13). Whereas clinicians agreed that providing alcohol-related information was useful, they expressed feeling insecure about when and how to provide it during the intervention:

*Clinician*: *I felt like wanting to give more*, *I*, *well*… *maybe I could have done so*, *right*, *but I would have liked to give more information [to the participant]*.

*Interviewer*: *About what?*

*Clinician*: *About the fact that*, *when she says that*, *in her opinion*, *a completely normal and non-problematic consumption is actually something relatively high; well I would have liked to provide her*, *well to give her this feedback*, *so that she could compare herself with respect to an actual norm*, *because without that*, *well maybe I should have allowed myself to do it right*, *but without doing it*, *it was almost as if I heard that she drank that but I normalized it as well or* … *[…]; but my question was whether I could do it while keeping a motivational stance*. (Q14).

The other half of the participants, on the other hand, reported no interest in receiving alcohol-related information. One of them explained for instance being more interested in sharing a “real discussion” with the clinician rather than getting information, while another participant considered that being told certain alcohol-related information could be upsetting: “*I think that you should be careful if you do*, *where you will give it*, *actually [talking about a list of safer-drinking strategies]*. *Because there are people*, *like myself for instance*, *I*, *if you say that to me I’m going to feel hurt*, *like well*, *hurt because you see me as an alcoholic… and I*, *I will take it badly*, *it will hurt me because I know that I’m not like that*, *and it irritates me*” (Q15).

Most participants reported that they valued evoking change in this specific situation. For example, one participant expressed that “*It feels good to see*, *well to be able to think further*, *after that [the emergency department experience]*, *because*, *well*, *I would never have thought about myself here*, *so well*, *I would never have thought about having to think about a change because I am here either*” (Q16). Congruent with participants’ reports, clinicians noted that participants appeared at ease when evoking change and that it stimulated reflection.

#### Participants’ and clinicians’ perceptions of the intervention’s utility

Participants commonly reported that “*it felt good*” to talk. One participant reflected: “*Well*, *it feels good to*, *maybe you don’t realize it at first but*, *it feels good to have the opportunity to talk*, *about*, *about what happened*, *yeah*, *it is always good to talk about* … *Keeping stuff to yourself isn’t good and if you can talk to someone else*, *it is always good”*“ (Q17).

Furthermore, most participants noted that the intervention made them think about alcohol use and related harm: “*It makes you think*, *always*, *and*, *well*, *it is not harmless*, *it’s a drug anyway*, *and even though it is accepted by society I mean*, *being able to find the right balance*, *well*, *being able to adapt your drinking*, *[…] avoiding having the glass too many*” (Q18).

Relatedly, a participant mentioned that the most useful part of the intervention was when they evoked change in their alcohol use. Clinicians’ perceptions of the intervention’s utility were somewhat more nuanced. On the one hand and in line with participants, both clinicians noted that the intervention made participants feel good and think of alcohol use and related harm. One clinician mentioned that the intervention led the participant to attribute causality between alcohol intoxication and ED admission: “*I tried to connect it*, *well*, *anyhow it is someone who fell down the stairs*, *while intoxicated*, *at the end of the party*, *and she initially said that it was just unfortunate*, *that it had nothing to do with alcohol… At the end of the interview*, *she said that*, *indeed*, *if she hadn’t drunk she wouldn’t have fallen*. *I thought that*, *in any case*, *maybe it was the use of it [the intervention*], *to have her verbalize it … and that she heard herself say*, *“Indeed maybe if I hadn’t drunk I wouldn’t have fallen*” (Q19).

On the other hand, talking about the intervention’s utility sometimes made clinicians think further. For instance, a clinician reported, *“Of course I think about what I could have done differently*, *about what the patient told me*, *and then about the fact that I don’t have the impression that she really moved on during this intervention*” (Q20).

### Conclusions of Round 1 and implications for next model iteration

Overall, the participants appreciated the intervention, felt free to speak, appreciated evoking change, and thought that the clinicians were attentive, kind, soothing, sincere, and non-judging. Clinicians’ perceptions were good overall, even if the hectic context of the ED was sometimes stressful and fostered some auto-pressure to conclude the intervention. The model was judged as feasible and the three steps (see Fig 2) made sense, even if in one intervention, the clinician did not apply the steps chronologically. Nevertheless, the third step appeared to be more challenging for clinicians, particularly when meeting with more contemplative participants. In response to this latter point, the next iteration of the model comprised a more developed description of Step 3 (i.e. Planning change: reflecting on next steps, make them concrete, and increase self-efficacy, ability and commitment to change).

This first experimental session round also stressed the question of providing information. Half of the participants were interested in receiving information, for instance to compare their drinking to others. Nevertheless, the other half preferred an open-ended discussion. The clinicians estimated that providing some kind of information (e.g., normative feedback, protective strategies) would have been useful in most interviews. However, they were rather insecure regarding when and how to provide information during the intervention. Providing some feedback, information, and/or advice, using MI techniques was thus formally introduced in the next iteration of the model. It was also held as a main topic for the next round, i.e. the experts’ consultation meeting.

### Round 2 –experts’ consultation

#### Topic 1—brief MI model overall

After presenting the intervention model in details, we showed the results of the qualitative feedback interviews from Round 1. Experts were also provided an intervention outline as a printed handout. Experts were asked to generate ideas and propositions to answer the following question: “Now that you have the intervention model in mind, what would you add and/or modify?”.

Overall, experts were supportive of the presented model, which they found feasible, acceptable, and theoretically sound. Experts generated and exposed 29 ideas to strengthen or improve the intervention model overall (see S2 Table). The most endorsed idea was to *add / focus on follow-up contact* (6 votes, score = 15). This topic was discussed in more details during the 3^rd^ part of the meeting (see below, Topic 3) but it was considered here that admission in the ED should be seen as a moment to engage patient into a discussion about alcohol use and consequences and that this discussion should be continued elsewhere or by other means. A similar idea was also suggested by an expert who thought about the intervention as a way to *make connections and engage people*, the target of the intervention not necessarily being to change or fix problems (2 votes, score = 5). Also in this direction of implementing the discussion and making it last, another idea was to *provide something patients can take with them*, such as a written change plan, a letter to themselves, a recording of the session, or other personalized item (3 votes, score = 5).

The second most endorsed idea, totaling the highest score (5 votes, score = 20) was to *provide a simple*, *structured*, *replicable model*. The current model was thought to be somewhat diffuse and complicated. Several experts advised to work more thoroughly on model presentation to make it more structured and easier to apply and replicate elsewhere.

The next most important idea (4 votes, score = 13) was to *give advice and directions* to the patient. This was considered a crucial ingredient of brief alcohol intervention. Experts added that such advice should be given within the same empathic and acceptant climate, and, as proposed in MI, after having authentically asked permission to offer advice and direction. This idea was close of another idea which was to *Give advice about avoiding coming back to ED* (1 vote, score = 5). It was also related to ideas about giving alcohol-related information, such as *Give age-matched normative feedback* (3 votes, score = 9), and *Give information about what they are in the ED for (information about alcohol intoxication)* (2 votes, score = 3). Related to the latter, one additional idea was to focus the discussion more on the *precipitating event* and *work on causal attribution of ED admission to alcohol intoxication* (3 votes, score = 11).

Another line of ideas, which was not anticipated and discussed for the model so far, was around social influence and social support. The most endorsed idea in this line (4 votes, score = 9) was to *work on social support*, mainly by adding a discussion about *where clients can get it*. Close to this, one idea was also to *work on peer influence* (1 vote, score = 2). There was also a discussion about the opportunity to *get someone else involved* (such as a peer, or a significant other (2 votes, score = 4) or *approach the group and not just the individual* (1 vote, score = 3). Even if actually involving other people in the discussion is not feasible in the study design, the experts pointed to the idea of discussing with the patient about how he/she could involve peers, friends, or significant others in their reflections and/or plans about change.

Then, two ideas were well received by experts but could not be implemented. The first was to include electronic elements in the intervention. One expert proposed to *give patients a tablet* with electronic intervention contents, such as short films and interactive information; the patient would talk for a while with the clinician, then work on the tablet, and talk again with the clinician (3 votes, score = 13). Another expert suggested to *consider computer-based intervention for less severe patients* (1 vote, score = 4). However, the study protocol included only face-to-face intervention and there was no budget to develop and pilot-test electronic contents. Another proposed idea was to *restrict age group to 18–24 years old* (2 votes, score = 6) in order to get a more homogenous population having similar alcohol-related problems, but again the study protocol was already planned and accepted by the study funder and the ethics committee with an age range of 18 to 35.

Finally, there were several other ideas, which were endorsed by only one expert or none, and were thus not considered as receiving sufficient consensus. These are listed in the table provided in S2 Table. Even if not considered among the most important by the experts, these ideas might still be of interest for further studies.

#### Topic 2—providing information

We first presented what type of information we thought about giving (e.g. age/gender tailored normative feedback, blood alcohol concentration estimation and relative consequences, estimated calorie intake, protective behavioral strategies, alcohol effects on health). Then, we presented results related to information giving from the qualitative feedback interviews from Round 1. Experts were then asked to generate ideas and suggestions to answer the following question: “How would you provide information?”, with sub-questions being “Which information? How to introduce it? When? (during BMI? after BMI in a leaflet? other way?) Systematically? (or according to particular need/circumstances?)”.

Experts generated and exposed 28 ideas or propositions. Among those, the idea voted as the most important (4 votes, score = 15) was to provide *highly individualized information*, which would be summed up in a *“letter” given to the client at the end of the intervention*. Providing individualized information was considered essential and preferred to generic information e.g. presented in a leaflet. For example, another idea was favorably rated (3 votes, score = 11): *What information*: *give tailored advice*, *with options*, *and specific not generic information*. Other types of information, also tailored to client’s situation, was recommended: *Information about intoxication (specific to client BAC) and normative feedback about intoxication* (2 votes, score = 9); or *Personalized “exercises” on options and strategies* (2 votes, score = 4). If information and advice were recommended to be tailored to clients, one expert noted that they should nevertheless be *simple and standardized to be replicable* (3 votes, score = 10). Even if not well supported in experts votes, there was also a large consensus during the discussion that *some information should be given to everyone*, *after having asked permission to provide it* (2 votes, score = 2); and that *minimal information should be given to everyone*, *with additional information depending on what people need or are interested in* (1 vote, score = 2).

The second most voted idea was related to the way of providing information to a population of young adults. One expert recommended using *no printed leaflet*, but *using smartphone to send messages tailored on what was discussed* (4 votes, score = 12). Similarly, another expert recommended to *multiply contacts and media (SMS*, *email*, *telephone)* (3 votes, score = 10). Several ideas, well received by other experts, were related to computerized ways of delivering information and advice: *Provide visually appealing information on a tablet*, *with graphs* (3 votes, score = 6); *Sending information from the tablet used during BMI to the client’s smartphone* (3 votes, score = 12). Experts considered that *computerized information would be friendlier*, by letting the *client decide what information is most useful* (3 votes, score = 6). In the same vein, but using technologic developments that were not in the scope of the present study, two proposals were supported by multiple experts and might thus be worth testing in another project. The first was to develop *computer interactive exercises using the MI technique ‘Elicit-Provide-Elicit’*, *tailored to target alcohol outcomes* (4 votes, score = 11). The second was to show a *video of a peer (age- and gender-matched) who experiences an appealing*, *positive story on something they can do since they are not intoxicated* (3 votes, score = 11), the idea here being to present a positive, desirable story and not a negative story (such as a car crash) which would not be effective after an admission in the ED related to alcohol intoxication.

Again, there were several other ideas, which were endorsed by only one expert or none, and were thus not considered as receiving sufficient consensus. These are listed in the table provided in S2 Table. Even if not considered among the most important by the experts, these ideas might still be of interest for further research.

#### Topic 3—intervention booster

As an introduction to this last topic, we presented the intended model for booster sessions. Booster were planned to be introduced during the initial BMI, while asking permission to contact participants again. Four modalities were proposed: 1) Send booster letters (sent within 1 week after BMI, with a goal to remind ED context, summarize discrepancy, hypothesized change, and potential change plan, remind date for phone call and treatment appointment if applicable; provide links to the website of the Alcohol Treatment Center and to an alcohol information website); 2) Phone booster sessions (systematically, pro-actively proposed during each BMI; phone call at 1 week, 1 month, 3 month, and 6 month); 3) Drinking goals monitoring using a Smartphone app (proposed in the case of a drinking change plan); 4) Accompany to specialized treatment (proposed in case of signs of severe alcohol problems). After presenting these options in details, we showed the results of the qualitative feedback interviews from Round 1. Experts were then asked to generate ideas and propositions to answer the following question: “Now that you have the intervention booster model in mind, what would add and/or modify?”

Overall, the experts found that the options presented were already a good range of options. A minority of experts debated the need to provide booster sessions at all, since they found that boosters were not necessarily cost-effective, that it is often difficult to reach people, and that some participants might not want to be contacted at that moment. Experts generated and exposed 19 ideas or proposals. Among those, the idea voted as the most important (6 votes, score = 22) was to opt for *electronic delivery of the booster letter* (e.g. via text message). This idea was consistent with other ideas such as *emailing pdf instead of sending letters and using text messages instead of phone calls* (1 vote, score = 4), using *multi modal delivery*, *including new technologies* (2 votes, score = 3), and using *online technologies*, *also to remind appointments*, *collect new data*, *etc*. (1 vote, score = 3), or using a *Smartphone application* (3 votes, score = 8). Additional ideas included suggestions of other forms of contact using new technologies such as text messages and videos, but were only marginally supported.

The second most important idea was to *ensure some follow-up contact*, for example health care contact in primary care soon after ED admission (5 votes, score = 18). The idea was that this contact in primary care would have a booster effect but might also provide an initial intervention for those not remembering what happened in the ED. If this idea was well supported by several experts, other cautioned about it since patients often not come back in person in a clinical setting. Another idea involving primary care was to *consider transferring referral to primary care* instead of specialized care (2 votes, score = 6). Most of the other ideas were related to booster content which should be *simple*, *feasible*, *and replicable* (4 votes, score = 15), *personalized* (3 votes, score = 8), *memorable* (2 votes, score = 6), *include fresh*, *individualized data* (4 votes, score = 14), but *limited*, i.e. less contact of better quality (4 votes, score = 10).

As for the other topics, there were several other ideas, which were endorsed by only one expert or none, and were thus not considered as receiving consensus for the present project. These are listed in the table provided in S2 Table. Even if not considered among the most important by the experts, these ideas might still be of interest for further studies.

### Conclusions of Round 2 and implications for next model iteration

Experts were largely supportive of the proposed intervention model, which they found feasible, acceptable, and theoretically sound overall. The ideas and propositions generated during this consultation meeting focused on two major points: ensure some follow-up contact (booster interventions), and provide information and advice. There was a consensus on favoring follow-up contact. The intervention in the ED was considered an opportunity to make connections and engage people in further care or follow-up discussion. Several options were discussed and most experts agreed that multiplying contacts and multi-modal delivery including new technologies (e.g. text messages, email, telephone) would be more convenient and adequate to contact a population of young adults. Experts also supported the idea of providing something that patients can keep, such as a “letter” summing up what was discussed and/or individualized information, which could be given to the client at the end of the intervention or electronically delivered. Other ways of offering follow-up contacts using new technologies were suggested but were beyond the scope of the present study. For our model, we retained the idea of multiplying contacts and multi-modal delivery by proposing up to 3 contacts through telephone, and a booster letter sent via email or by mail according to patient choice. This letter would also contain links to our website, which contains information on alcohol use and consequences, as well as help and treatment offers.

The second main point was related to advice and information. There was a consensus that some information should be given to everyone. Minimal information could be given to everyone, with additional information depending on what people need or are interested in. Information and/or advice should be tailored to the patient. Several ideas highlighted the importance of giving information about what patients are in the ED for (information about alcohol intoxication) and focusing the discussion on the precipitating event and the causal attribution of ED admission to alcohol intoxication. Experts also insisted on how to provide information, i.e. by asking permission to offer it, by using the same empathic and acceptant climate as proposed in MI, and by providing directions, advice, or strategies with options (and discussion of these options to capture which could be adequate). All these points were retained and added in our next intervention model.

A third, unexpected point caught our attention: the work on social support. Our intervention was initially developed in an individual perspective, taking into account mainly intrinsic motivation. We were therefore attentive to the idea brought forward by several experts around working on social support, getting peers or significant others involved, or approaching the group and not just the individual. Since actually involving other people in the discussion was not feasible in our model, we added the idea of discussing with the patient about how he/she could involve peers, friends, or significant others in their reflections and/or plans about change.

Finally, there was a strong consensus on the need to describe a simple, structured, and replicable model. We retained this suggestion and further refined our model in an intervention manual, encompassing the main intervention principles and content.

### Round 3 –experimental sessions

Similar to Round 1 evaluation, categories yielded in this second analysis reflected participants’ and clinician’s perceptions of the intervention’s feasibility, acceptability and utility.

#### Participants and clinicians’ perceptions of the intervention feasibility

*Suitability of the intervention in the ED*. Similar to Round 1 evaluation, participants and clinicians uniformly considered that the ED was suitable for the intervention. Most participants were in favor of receiving the intervention in the ED instead of doing it a few days later. For instance, one participant explained: “*Well I would prefer doing it now*, *because*, *actually the way I feel now*, *I mean*, *I regret I went out*, *I mean*, *having taken stuff before*, *thus I would prefer doing it now than waiting one day and having had a rest*” (Q21).

Similarly, both participants and clinicians reported that doing the intervention in the ED while participants still feel “*shocked*” and/or “*confused*” represents a potential change trigger. For instance, a clinician reported that doing the intervention in that specific moment may strengthen participants’ discomfort and, as a consequence, their motivation to avoid coming back to the ED while intoxicated. That said, similar to Round 1, both clinicians and participants considered participants’ state as a potential disadvantage of doing the intervention in the ED. Participants commonly reported feeling “*tired*,” “*shocked*” or “*confused*” and therefore sometimes not able to deepen the discussion and keep a clear memory of its content. Clinicians shared this point of view while underlying the importance of clinical boosters: “*Well I think that it is not possible to do everything in a single intervention in the emergency department*, *in fact*, *because the person is still in a state*, *well*, *shocked*, *there is probably still alcohol involved there*, *there is emotion […] I think that*, *well*, *talking again about these things later on*, *when the person has been able to think*, *well*, *I think it is*, *it would be much*, *it would be good*, *effective*” (Q22).

*Perceptions of the intervention’s model applicability*. Similar to Round 1, both clinicians indicated that the model and its three steps were overall applicable in the ED setting. Next, similar to the previous evaluation, clinicians uniformly reported encountering difficulties in deepening the discussion at some points during the intervention, as well as in addressing concrete plans to change.

#### Participants’ and clinician’s perceptions of the intervention’s acceptability

Corroborating findings from Round 1, all participants reported feeling at ease and experiencing the intervention well. Unlike Round 1 however, the intervention’s length was consistently perceived as adequate, and even as an added value. For instance, one participant reported: “*the clinician let me express myself […] I did not feel stressed…well*, *how shall I put it… restricted by time or anything*” (Q23), whereas another mentioned appreciating that the clinician took time to understand her.

We also investigated participants’ feelings about potential intervention booster sessions (this was not done in Round 1). All participants were interested in receiving a letter summing up the discussion with the clinician. Participants commonly explained that it would help them remember what happened that brought them to the ED, as well as the content of the discussion. In the same vein, participants evenly accepted to be called by the clinician one week later (i.e., 1-week booster). Some participants mentioned appreciating feeling “*helped*,” whereas for others, boosters indicated a commitment of the clinician: “*I think this is good […] it actually shows an investment between*, *well*, *both people [the clinician and the participant]*” (Q24).

*Participants’ perceptions of the clinicians*. All participants’ perceptions of the clinicians were positive in this round. Similar to round 1, participants reported feeling “*at ease*” with the clinicians and even “*trusting them*”. They were commonly perceived as “*kind*” and “*understanding*.” Other positive perceptions held by some participants included the fact that clinicians were attentive and showed interest in the participants’ discourse.

*Participants and clinicians’ perceptions of the intervention’s content*. Consistent with Round 1, the intervention’s content was well received among participants. At least two participants mentioned that the clinicians asked them “*the good questions*.” One of them went on explaining he appreciated that the intervention’s content was personalized: “*It was not like it was written questions*. *I mean that*, *if I say something*, *she will ask me a question about it*” (Q25).

Following Round 1 and Round 2 amendments to the intervention model, alcohol-related information was systematically provided in this round. Most participants had a positive perception of the information they received. Information was commonly described as “*interesting*” or “*complete*”. That said, receiving alcohol-related information was also perceived as a “formality” and its utility was commonly questioned. In fact, participants mentioned almost uniformly that they did not learn anything new: “*Well*, *I already knew that there were plenty of risks [laughing]*, *so I didn’t*, *I didn’t think too much*, *I already knew*” (Q26). This point of view was corroborated by clinicians who reported tailoring the information content to the participants’ knowledge and reactions: “*I asked her somewhat if she knew [alcohol-related] effects*, *and then…*, *and then*, *we talked a little bit about that*. *I didn’t go into lots of details because I didn’t have the impression that she needed more than she already had*” (Q27). The clinicians explained that providing information about alcohol-related consequences happened naturally during the intervention, whereas the way of introducing them varied across interventions. For instance, a clinician explained: “*After I asked him what he thought about the fact of ending up here*, *in the ED*, *and so on*, *well I thought it was a good time to do that and well he was ok with it*, *so yes […]*. *I asked him if he actually agreed to talk about alcohol-related consequences and when he said yes*, *I started by asking him what he knew […] And so he spontaneously gave you*, *well told you a bit the risks he knew about alcohol […] and so I asked him if he was ok that I complete what he said*, *while of course validating what he said*” (Q28).

#### Participants’ and clinician’s perceptions of the intervention’s utility

Most participants perceived the intervention as useful. Similar to Round 1, participants frequently reported that “*it felt good talking*.” Echoing this point of view, a clinician reflected, “*I have the impression that the intervention also did him a lot of good*. *Well*, *as he talked a lot*, *I think he really needed to unwind and I think it made him feel good that there was somebody who*, *who could listen to him too*” (Q29). Both clinicians and participants commonly reported that the intervention made them think. Some participants added that it helped them gain a better understanding of what brought them to the ED, and a better understanding of their alcohol use and related harm more broadly. For instance, a participant reflected: “*What helped me a bit*, *was precisely to understand why I am here [in the emergency department] and*, *why I did that*” (Q30); whereas another reported: “*it made me understand that you really need to pay attention to what you drink […] you need not to drink too much either*, *too fast*, *you need above all to keep hydrated*, *to eat well*” (Q31). On the contrary, two of the 6 participants reported that they did not find the intervention useful. Specifically, one participant explained that “*he did not gain anything from it*,” whereas the other participant explained that she did not need the intervention to decide to make a change: “*Well*, *anyway I was already disgusted with myself*, *by myself*, *that I would not go out*, *so it’s not the intervention that influenced it*” (Q32).

### Conclusions of Round 3 and implications for the final model

Similar to Round 1 evaluation, participants and clinicians uniformly considered that the ED was suitable for the intervention. The recency of the shock related to ED admission was commonly considered a potential change trigger. However, confusion and tiredness were also mentioned as potential disadvantages, preventing from deepening the discussion and keeping a clear memory of its content. This barrier underlined the importance of intervention boosters. Participants consistently accepted to be called back by the clinician. All participants were also interested in receiving a letter summing up the discussion. Participants commonly explained that it would help them remember what happened, what brought them to the ED, as well as the discussion’s content. It was also perceived as indicating a commitment of the clinician in the relationship being built. Consequently, the booster letter (sent by mail or email) and the phone calls were established in the final model.

Following Round 1 and 2 amendments to the intervention model, alcohol-related information was systematically provided in round 3. The clinicians happened to provide information about alcohol-related consequences naturally during the intervention, whereas the way of introducing them varied across interventions. Most participants had a positive perception of the information received, which was commonly described as interesting and complete. Nevertheless, receiving alcohol-related information was also perceived as a “formality” and its utility was commonly questioned since almost all participants mentioned that they did not learn anything new. According to this feedback and to experts’ consultation in Round 2, we further discussed this major point in our working group. In the final model, we specified that minimal information should be systematically provided to all participants in two ways: 1) when introducing the intervention, by specifying that we try to reach out every young adults admitted in the ED while intoxicated since previous studies showed that being admitted intoxicated was related to higher likelihood of being readmitted, of developing alcohol and other drugs problems, or of having psycho-social problems; 2) by providing links to alcohol-related information website in the booster letter. In addition, clinicians should pay attention to patients’ knowledge and systematically address the following topics when needed: 1) causal attribution of ED admission to alcohol use, 2) adjust distorted perceptions, banalization, and misbeliefs about alcohol use and consequences; and 3) suggest change options or strategies if necessary. While doing so, clinicians have to use MI techniques [14], i.e. Elicit (patient’s prior knowledge)–Ask permission (to give information)–Provide (information, advice, strategies options)–Elicit (what do patient think about it?).

Otherwise, all participants’ perceptions of the clinicians were positive in this round. Participants confirmed feeling “at ease” and understood, and trusting clinicians, whom they found kind, attentive, and showing interest in their discourse. Consistent with Round 1, participants appreciated the intervention’s content and found that the clinicians asked them “the good questions”. They also appreciated that the intervention’s content was personalized. Unlike Round 1, the intervention’s length was consistently perceived as adequate, and even as an added value (i.e. providing enough time to express themselves and to be understood). On other hand, clinicians felt again that the intervention model strategies and its three steps were applicable overall. However, similar to the previous evaluation, difficulties in deepening the discussion at some points, as well as in addressing concrete plans to change were mentioned. We believe that the possibility of offering information, advice, and change strategies options as described above might also have an effect with patients having difficulties in deepening the discussion and/or addressing concrete plans to change. Booster interventions might also be effective, particularly when difficulties in deepening the discussion are related to confusion induced by intoxication and/or emotional status.

Finally, most participants perceived the intervention as useful. Similar to Round 1, participants frequently reported that “it felt good to talk”. The intervention made them think and helped them gain a better understanding of what brought them to the ED, and a better understanding of their alcohol use and alcohol-related harm more broadly. Nevertheless, some of the participants reported that they did not find the intervention useful. When considering these critical remarks, we further refined two parts of our final intervention manual. First, we intensified the importance of relational factors to build a significant relationship, notably through showing curiosity and empathy. Secondly, we further developed the first step of the model where the clinician explores the current situation and puts it into perspective with important things in life and values. Using relational factors and reflective listening, the clinician takes the time to really get to know who the patient is, what are their current situation, their values, and the meaning they give to it.

## Discussion

The present article aimed to develop and pre-test a new motivational intervention model for young adults admitted in the ED with alcohol intoxication using an iterative qualitative process. This process generated 20 semi-structured interviews and 76 experts’ propositions. It allowed us to test the intervention model in the real world and to collect clinicians’ and patients’ feedback on their experience, as well as to theoretically refine our model. At each round, data were analyzed and discussed to update our intervention model. A schematic outline of the final model is provided in Fig 2. The final intervention manual is available, in French, on request to the first author.

Discussion of specific study results and related conclusions are provided at the end of each round and, consequently, we do not provide further discussion of study findings here. Overall, this iterative process of intervention development offered a strong support for the acceptability and feasibility of our model to address alcohol use and related problems with young adults in the hectic context of the ED and following an alcohol intoxication. As observed in similar recent studies [e.g. 68–71], qualitative interviews provided detailed insights regarding the acceptability, feasibility and value of the pretested intervention, while offering information necessary to enhance its components. As noted by Darker, Sweeney [71], there is a paucity of research that describes the process of tailoring interventions, and using qualitative methods proved to be valuable to identify strengths and necessary modifications to the model before its implementation.

Despite the above-noted strengths, this study has also some limitations. In rounds 1 and 3, interventions were conducted by the same two female psychologists. If this provided higher internal consistency, it obviously decreased variability in clinicians’ characteristics and within-session behaviors; and results might have been biased by therapist effects [72, 73]. In these same rounds, analyses might be limited to some extent since the same interviewer conducted all semi-structured interviews and analyses. Nevertheless, analyses were reviewed and discussed with the first author on a regular basis, as well as discussed by the whole working group at the end of each round, thus providing in-depth triangulation of data and findings. Round 2 was limited by time and experts’ availability. Throughout all rounds, we had to balance between the richness of the analyses of the data collected and the burden of this collection process. This burden notably includes the length of semi-structured interviews, their transcription and analyses, as well as mobilizing 9 international experts during a 4-hour meeting and analyzing the resulting numerous inputs.

Nonetheless, by this careful and rigorous iterative process, we were able to develop, pretest, and refine an intervention model, of which the efficacy will be tested as the next phase of the project. This is done using a randomized controlled trial that is ongoing and registered in the ISRCTN registry (http://www.isrctn.com/ISRCTN13832949). Results are expected at the end of 2020. A further phase of the project will be to evaluate the mechanisms of the intervention effects using a moderated mediation framework [74], including observational coding process, as well as qualitative analyses using semi-structured interviews with randomly selected patients after the 1-month and 12-month follow-up questionnaire.

## Supporting information

S1 File Interview grids for semi-structured interviews. (PDF)

S1 Table Original quotes in French. (PDF)

S2 Table Ideas generated by experts and experts’ scoring of these ideas using the nominal group technique. (PDF)
