# Supplementary material for: Developing a brief motivational intervention for young adults admitted with alcohol intoxication in the emergency department – Results from an iterative qualitative design
Source: PLoS One. 2021 Feb 8;16(2):e0246652. doi: 10.1371/journal.pone.0246652 (PMC7869998; doi:10.1371/journal.pone.0246652)
Supplement: S1 File — (PDF) [file pone.0246652.s001.pdf]

S1 File

Interview grids for semi-structured interviews

Contents

Version for interviews with the Clinician – translated to English 2

Version for interviews with the Patient – translated to English 5

Original French version for interviews with the Clinician 8

Original French version for interviews with the Patient 11

Version for interviews with the Clinician – translated to English

PRELIMINARY INSTRUCTIONS		***Aim***
- The goal of this interview is to find out how you felt about the intervention you just conducted and to see how you experienced the application of the intervention model with the patient you met.<br>- The main goal of this interview is to improve the intervention model based on your feedback.<br>- In order to save time and to cover all the specific points, I might sometimes redirect our discussion.<br>- We are interested in some specific points and sometimes the questions might look similar.<br>- As discussed, the interview will be recorded. The recording will only be used to transcribe the interview and analyze its content. It will not be shared elsewhere.<br>- Before we start, do you have any questions?		*Contextualize the frame and goal of the interview in particular to reassure the clinician (evaluation of the intervention and not of the clinician's performance).*<br><br>*Anticipate the structured nature of the interview and the allocated time.*
**THEMES**	**PRIMARY AND SECONDARY QUESTIONS**	***Aim***
EXPERIENCE OF THE INTERVENTION		
➔ *During your last intervention….*		
Overall experience (icebreaker)	**What was your experience of the session?**<br>**Tell me how the intervention went.**<br><br>*Inform that we will now move on to more specific questions.*	*Evaluate feeling of comfort when using the model (free topic, context or content)*
Context	**Where did the last intervention take place?**<br>**Where were you in the room?**<br>➢ Sitting, standing? Chair, bed?<br>**Where was the patient?**<br>➢ Bed, chair? Sitting, standing?<br>**Could you conduct the intervention in full, without interruption?**<br>➢ Stopped by clinical care? Other clinical staff visit? Patient status? Confidentiality?<br>**How did you feel in this context?**	*Verify feasibility of the intervention in the ER context.*<br>*Verify which contextual/logistical modalities are the most adequate.*
Applying the model: motivational spirit and relational factors	**How would you describe this intervention in terms of motivational spirit and relational factors?**<br>➢ Empathy, acceptance, collaboration?<br>➢ Obstacles, facilitators?<br>**To what extent did this intervention allow you to create a relationship with the patient?**<br>➢ Key factors?	*Assess how motivational spirit was applied in a specific context.*<br>*Bring to light the potential difficulties of applying motivational spirit.*<br>*Verify if and how a single intervention in the ER makes it possible to create a significant relationship with the patient.*

Applying the model overall	**If you think about the model in general, how did this intervention go?**<br>➢ Easiness? Difficulties? Taking some liberties with the model? Why? Constraints related to the model? Why?<br>➢ Content?	*Asses the overall relevance of the model and its applicability in the field.*<br>*Assess to what extent the principles and contents of the intervention are applicable to a variety of patients.*<br>*Explore which particular situations make applying the model problematic.*
Applying the model: intervention outline and the 3 steps	**How did this intervention go, more specifically regarding its structure and its rhythm?**<br>➢ implementing the 3 steps<br>➢ Why?<br>➢ Obstacles, facilitators?	*Assess the applicability of the 3-step model.*<br>*Explore situations that might challenge how the 3 steps are implemented.*
Applying the model: causal attribution	**How was it like when you talked about the link between ER admission and alcohol use?**<br>➢ Who broached this topic and how?<br>➢ How did you perceive the patient at this moment?<br>➢ Obstacles, facilitators?	*Assess how causal attribution is applied.*<br>*Explore situations that might challenge how causal attribution is discussed.*
Applying the model: giving information (about alcohol related risks)	**What information (about alcohol related risks) did you give the patient?**<br>➢ At which moment in the interview?<br>➢ Why this kind of information?<br>➢ For what purpose?<br>**How did the patient react when you gave this information?**<br>➢ How did you perceive the patient at this moment?	*Assess how giving information is applied and if it is relevant.*<br>*Highlight which information are pertinent in which situation.*<br>*Assess how the clinician perceived the patient when giving information.*
Applying the model: giving advice	**To what extent did you give the patient advice?**<br>➢ If yes, which?<br>➢ Why?<br>**How did the patient react when you provided advice?**<br>➢ How did you perceive the patient at this moment?<br>➢ What did he/she said about it?	*Assess how giving advice is applied and if it is relevant.*<br>*Assess how the clinician perceived the patient when giving advice.*
Applying the model: change talk	**What was it like to address the topic of change with the patient?**<br>**How do you think the patient experienced this part of the interview?**<br>➢ deepen exploration of the current situation and establish the intervention in the long run<br>➢ Resistance? Comfort?	*Verify the relevance of going beyond exploration of the current situation during an intervention.*<br>*Explore the feeling of comfort of both the clinician and the patient when exploring change and planning.*

Applying the model: planning	**How was the planning phase (concrete planning)?**<br>➢ Desired change?<br>➢ Goals?<br>➢ Obstacles, facilitators?<br>**How did the patient react during this phase?**	*Assess how planning is implemented.*<br>*Highlight the possible difficulties during the planning phase*<br>*Assess how the patient reacted during this phase.*
Applying the model: influence of peers and relatives	**To what extent did you discuss the patient's social network, relatives and peers?**<br>➢ Support/obstacle to achieve the change plan?<br>**How do you think the patient felt about talking about his/her peers/relatives?**	*Assess application and relevance of peer/relative influence*<br>*Assess how the patient reacted at this moment of the interview.*
Applying the model: taking time	**Did the intervention allow you to engage the patient in a reflection that will be sustainable in time?**<br>➢ Future planning, change goals<br>➢ Follow-up terms	*Verify if a single intervention in the ER allows initiating future plans*
Suggestions for change	**What do you think the patients would need?**<br>**According to you, what adaptations of the model would allow going in this direction?**	*Explore clinician's frustrations related to the intervention and the patients' needs.*<br>*Highlight potential suggestions of adaptation of the model's content.*
CLINICAL FOLLOW-UP		
Follow-up modalities	**What follow-up did you and the patient agree on?**<br>➢ According to you, what did the patient think about it?<br>➢ According to you, what are the patient's needs and expectations?<br>**To what extent do you think that sending a written report of the intervention would be relevant in this situation?**	*Explore follow-up modalities that would be the most adapted to follow-up with an intervention in the ER setting.*<br>*Complement / adapt the intended modalities.*
CLOSING	**Is there anything else you would like to share about this intervention before we stop this interview?**	*Elicit possible additional elements that were not directly raised by the interviewer.*<br>*Smoothly close interview by letting the interviewee speak.*

Version for interviews with the Patient – translated to English

INTRODUCTION / PRELIMINARY INSTRUCTIONS		*Aim*
- Hello, my name is ___. I'm working for the Alcohol Treatment Center as a research collaborator.<br>- As my colleague has told you, I would like to take a few moments to talk with you. The goal of this interview is to understand your feelings about the intervention that you've just had. Your opinion is particularly important to allow us to strengthen patient care.<br>- The interview should last around 20 minutes.<br>- In order to save time and to allow covering a certain number of points, I might sometimes redirect our discussion if necessary.<br>- You may find some of my questions somewhat repetitive along the interview; I apologize for that in advance.<br>- Just like the previous interview, this discussion will be recorded. The recording will only be used to transcribe the interview and analyze its content. It will be destroyed at the end of the research.<br>- Before we start, do you have any questions?		*Contextualize the frame and goal of the interview (differentiate the research goal of this interview and the clinical goal of the intervention before).*<br>*Anticipate the structured nature of the interview and the allocated time.*<br><br>*Answer potential additional questions.*
**THEMES**	**PRIMARY AND SECONDARY QUESTIONS**	***Aim***
ICEBREAKER		
Motivation to participate and understanding of the context	**Why did you accept to take part in this interview?**<br>→What did you think about this proposition?<br>→ Which elements of the presentation made you decide to participate?<br>**In general, how did this interview go?**<br><br>*Inform the patient that we will now move on to more specific questions.*	*Initiate the discussion.*<br>*Explore patient's initial motivations.*<br>*Explore patient's understanding of the clinical context and clinician's objectives.*
EXPERIENCE OF THE INTERVENTION		
Context	**Can you describe the concrete context (the "scene") of the interview for me, as if you were painting a picture of it?**<br>→ What were you doing when the clinician asked to talk with you?<br>→ Particular moment in patient's clinical care? Sleeping, awake? During a clinical act?<br>→ Where? Clinician-patient setup? Sitting, lying down?<br>**According to you, what context would have been ideal?**<br>→ Other clinician, ER physician?<br>→ By phone? At home?	*Verify intervention feasibility in the ER context.*<br>*Verify which contextual/logistical modalities are the most adequate.*

	→ After a few days?<br>→ Other context then ER?	
General experience	**In general, what was your experience with this interview?**<br>→ At ease? Appropriate? Length? Intimacy?	*Evaluate feeling of comfort during the intervention in terms of context and/or content.*<br>➔ *Encourage a maximum of spontaneous answers at this time of the interview.*
Intervention experience: motivational spirit and relational factors	**What would you say about the general attitude of the clinician during the interview?**<br>→Empathic, non-judgmental attitude?<br>**To what extent did you feel at ease with this person?**<br>→ Confidence, understanding, listening.	*Explore the patient's perception of the clinician's attitude.*<br>*Assess if the motivational spirit match the patient's expectations.*
Intervention experience: taking time	**What would you say about the quality of the interview?**<br>→ Enough time? In-depth exploration of individual situation?	*Verify if a single intervention in the ER allows creating a significant relationship.*
Intervention experience: content / intervention outline (3 steps)	**If you were to summarize the content of the interview to a friend, what would you say?**<br>→ Specific topics addressed? Beginning? End?<br>→ Different steps within the interview?<br>→ Specific goal of the interview?	*Assess the perception of an intervention in various steps (interview dynamics, progression).*<br>*Assess the perception of intervention's purpose.*
Intervention experience: causal attribution	**Did the discussion bring you to make links between your drinking and your admission to the ER?**<br>→ How was the topic introduced?<br>→ By whom and at what moment during the interview?	*Assess the perception of causal attribution.*
Intervention experience: information about alcohol related risks	**During this interview, what information did you receive about alcohol related risks?**<br>→ What are your thoughts about it?<br>→ What will you do with it?<br>→ How did you feel at this moment of the interview?	*Assess the application and relevance of giving information*
Intervention experience: advice	**To what extent did the clinician give you advice about your drinking?**<br>→ If so, what advice were you given? What do you think about it? How did you feel?<br>→ If not, what kind of advice would you think might be useful?	.<br>*Assess the application and relevance of giving advice*
Intervention experience: change talk, planning	**To what extent did you talk about things you would like to put in place in your life?**<br>→ Go beyond exploration of current situation.	*Verify the relevance of going beyond exploration of the current situation and planning the future during an intervention.*

	→ What kind of changes were discussed?<br>**How did you experience this part of the discussion?**<br>→ Resistance? Stress? Felt intrusive?	*Explore the feeling of comfort while evoking change.*
Intervention experience: influence of peers and relatives	**To what extent did you talk about your relatives and peers (friends, family) during the interview?**<br>→ Support/obstacles to implement plan?<br>→ What do you think about that?<br>**How did you experience this part of the discussion?**	*Assess whether it is relevant to involve the social network during the intervention (without them being present)*
Impact of the intervention	**Overall, what are the things that you discussed during this interview that have been/will be useful for you or that you appreciated?**<br>→ And those not useful, not appreciated?<br>→ Did anything bring you to see your situation differently?<br>**How do you think that you will be thinking about this interview tomorrow?**	*Assess the immediate impact of the intervention.*<br>*Highlight the strengths and weaknesses in terms of felt usefulness.*
CLINICAL FOLLOW-UP		
Follow-up modalities	**What follow-up did you and the clinician agree on?**<br>→ What are your thoughts about that?<br>→ Would you have any suggestions/desires/expectations?<br>**What would you think about receiving a written report of the interview?**	*Explore follow-up modalities that would be the most adapted.*<br>*Complement / adapt the intended modalities.*<br>*Verify the interest of following-up the discussion and opportunity to initiate planning the future.*
Suggestions for change	Content:<br>**Ideally, what would you have wished to happen during the interview with the clinician?**<br>→ Would you have liked to do a quantitative evaluation of your drinking and receive feedback about it?	*Assess patient's expectations in terms of clinical care and of contents.*<br>*Explore contextual elements and contents that are unsuitable for patient's expectations/status/capacities.*
CLOSING QUESTION	**Is there anything else you would like to share before we stop this interview?**	*Bring out possible additional elements that were not directly raised by the interviewer.*<br>*Smoothly close interview by letting the interviewee speak.*

Original French version for interviews with the Clinician

**CONSIGNES PREALABLES**		***Intentions***
- L'idée de cet entretien est de connaître votre sentiment sur le déroulement de la dernière consultation que vous avez menée et de voir comment vous avez vécu l'application de l'IM avec le dernier patient que vous avez rencontré.<br>- L'objectif principal de l'entretien est d'améliorer l'IM à partir de votre feedback.<br>- Afin de ne pas vous prendre trop de temps et de couvrir un certain nombre de questionnements spécifiques, je me permettrais parfois de rediriger un peu notre conversation,<br>- Nous nous intéressons à certains aspects spécifiques et parfois les questions se ressemblent un peu.<br>- Comme convenu, nous allons enregistrer l'entretien. Cet enregistrement vise uniquement à retranscrire les entretiens afin d'analyser leur contenu et ne seront pas partagés.<br>- Avez-vous une question avant que nous commencions?		*Contextualiser le cadre et l'objectif de l'entretien, ceci notamment afin de rassurer le clinicien (évaluation de l'outil et non de la prestation du clinicien).*<br><br>*Anticiper une conduite structurée de l'entretien et le temps imparti.*
**THEMES**	**QUESTIONS & RELANCES**	***Intentions***
EXPERIENCE DE L'IM		
➔ Lors de la dernière consultation….		
Vécu général (entrée en matière)	**Comment avez-vous vécu cette dernière IM?**<br>**Dites-moi un peu comment s'est passé l'entretien?**<br><br>*Annoncer que nous allons maintenant passer à des questions plus spécifiques.*	*Évaluer le sentiment de confort des cliniciens dans l'utilisation du modèle (thématique libre, contexte ou contenu)*
Contexte	**Où a eu lieu la dernière consultation?**<br>**Où vous êtes-vous placé dans la salle?**<br>→ Assis, debout? chaise, lit?<br>Où était le patient?<br>→ Lit, chaise? couché, assis?<br>**L'IM a-t-elle pu être conduite en entier et sans interruption?**<br>→ Soins cliniques? visite d'un autre professionnel? état du patient? confidentialité?<br>**Comment vous êtes-vous senti dans ce contexte?**	*Vérifier la faisabilité des IM au Service des urgences.*<br>*Vérifier quelles modalités contextuelles/logistiques sont les plus adéquates.*
Application du modèle: l'esprit motivationnel et les facteurs relationnels	**Et comment situez-vous cette IM par rapport à l'esprit motivationnel et les facteurs relationnels sur lesquels il repose?**<br>→ Empathie, acceptance, collaboration?<br>→ Obstacles, facilitateurs?	*Évaluer l'application de l'esprit motivationnel dans un contexte spécifique.*

	**Dans quelle mesure cette intervention vous a permis de créer une relation avec le patient?**<br>→ Facteurs déterminants?	*Mettre en évidence les éventuelles difficultés à la bonne application de l'esprit motivationnel.*<br>*Vérifier si et comment une IM unique dans le cadre des urgences permet de créer une relation significative.*
Application générale du modèle	**Si on pense au modèle général de l'intervention, comment s'est passée cette consultation?**<br>→ Facilités? difficultés? libertés par rapport au modèle à disposition? pourquoi? contraintes par rapport au modèle? pourquoi?<br>→ Contenu?	*Evaluer la pertinence générale de l'outil et son applicabilité sur le terrain.*<br>*Evaluer dans quelle mesure les principes et contenus de base de l'IM peuvent être appliqués avec une diversité de patients.*<br>*Explorer les situations qui rendent l'application du modèle problématique.*
Application du modèle: le schéma d'entretien et ses 3 étapes	**Comment s'est passée cette dernière IM plus spécifiquement en termes de structure/rythme?**<br>→ Application des 3 étapes?<br>→ Pourquoi?<br>→ Obstacles, facilitateurs?	*Evaluer l'application d'un modèle en 3 étapes.*<br>*Explorer les situations qui rendent l'application des 3 étapes problématique.*
Application du modèle: Attribution causale	**Comment s'est passé le moment où vous avez parlé du lien entre l'admission aux urgences et l'alcoolisation?**<br>→ Qui l'a abordé et comment?<br>→ Comment avez-vous senti le patient à ce moment de l'entretien?<br>→ obstacles, facilitateurs?	*Evaluer l'application de l'attribution causale.*<br>*Explorer les situations lors desquelles l'attribution causale est difficile à réaliser*
Application du modèle: Informations (par rapport aux risques liés à l'alcool)	**Quelles informations (sur les risques liés à l'alcool) avez-vous données au patient?**<br>→ À quel moment de l'entretien?<br>→ Pourquoi tel type d'information?<br>→ Dans quel but?<br>**Comment le patient a réagi lorsque vous lui avez donné ces informations?**<br>→ Comment avez-vous senti le patient?	*Evaluer l'application et la pertinence de donner de l'information.*<br>*Mettre en évidence quelles informations sont plus pertinentes que d'autres selon les situations.*<br>*Evaluer comment le clinicien a perçu le patient au moment de donner de l'information.*
Application du modèle: Conseils	**Dans quelle mesure avez-vous donné des conseils au patient?**<br>→ Si oui, lesquels?<br>→ Pourquoi?<br>**Comment le patient a réagi lorsque vous avez donné ces conseils?**<br>→ Comment avez-vous senti le patient?<br>→ Qu'en a-t-il dit?	*Evaluer l'application et la pertinence du fait de donner des conseils.*<br>*Evaluer comment le clinicien a perçu le patient lorsqu'il lui a donné des conseils*

Application du modèle: le discours changement	**Comment avez-vous vécu le fait d'aborder le changement avec le patient?**<br>**Et comment pensez-vous que le patient a vécu cette partie de l'IM?**<br>→ Dépassement de l'exploration de la situation présente et instauration de l'IM dans la durée.<br>→ Résistances? confort?	*Vérifier la pertinence de dépasser l'évaluation de la situation actuelle dans le cadre de l'IM.*<br>*Explorer le sentiment de confort du clinicien et du patient dans l'exploration du changement et la projection.*
Application du modèle: Planification	**Comment s'est passée la planification plus concrètement?**<br>→ Changements souhaités?<br>→ Objectifs?<br>→ Obstacles, facilitateurs<br>**Comment avez-vous senti le patient à ce moment de l'entretien?**	*Evaluer l'application de la planification.*<br>*Mettre en évidence les éventuelles difficultés à l'application de la planification.*<br>*Evaluer comment le patient a réagi à ce moment-là de l'entretien?*
Application du modèle: Influence de l'entourage	**Dans quelle mesure avez-vous parlé de l'entourage du patient?**<br>→ Soutien/obstacle pour la réalisation du changement?<br>**Comment pensez-vous que le patient a vécu le fait de parler de son entourage?**	*Evaluer l'application et la pertinence d'aborder l'influence de l'entourage.*<br>*Evaluer comment le patient a réagi à ce moment-là de l'entretien.*
Application du modèle: prendre le temps	**L'IM vous a-t-elle permis d'inscrire la réflexion du patient dans la durée?**<br>→ Projections, objectifs changement<br>→ Modalités de suivi	*Vérifier si une IM unique dans le cadre des urgences permet d'initier une projection dans le temps avec le patient.*
Suggestions de changement	**De quoi pensez-vous que les patients ont besoin?**<br>**Quelles adaptations du modèle permettraient d'aller dans ce sens selon vous?**	*Explorer les frustrations des cliniciens par rapport à la consultation et aux besoins du patient.*<br>*Mettre en évidence les éventuelles suggestions d'adaptation du contenu du modèle.*
SUITE PRISE EN CHARGE		
Modalités de suivi	**Qu'avez-vous convenu comme suite?**<br>→ Selon vous, qu'en pense le patient?<br>→ Selon vous, quelles sont les attentes et les besoins du patient?<br>**Dans quelle mesure pensez-vous que qu'envoyer un compte-rendu est pertinent dans cette situation?**	*Explorer les modalités de suivi les plus adaptées pour donner suite à une IM dans le cadre des urgences.*<br>*Compléter/adapter les modalités de suivi envisagées.*
CLOTURE	**Y a-t-il autre chose que vous souhaitez partager concernant cette consultation avant que nous terminions cet entretien?**	*Faire ressortir d'éventuels éléments complémentaires non abordés directement par l'enquêteur.*<br>*Terminer l'entretien de façon douce, en laissant la parole à l'interviewé.*

Original French version for interviews with the Patient

<table>
<tr><th colspan="2">INTRODUCTION/CONSIGNES PREALABLES</th><th>Intentions</th></tr>
<tr><td colspan="2">- Bonjour, je suis X. Je travaille aussi au Service d’alcoologie, comme chercheuse.
- Comme vous l’a expliqué ma collègue, je vous propose de discuter encore un moment avec vous. L’idée est de connaitre votre sentiment sur l’entretien que vous venez d’avoir. Votre avis est particulièrement important pour nous permettre d’améliorer la prise en charge des patients.
- L’entretien devrait durer environ 20’.
- Afin de ne pas vous prendre trop de temps et de couvrir un certain nombre de questionnements, je me permettrais parfois, si cela se voit nécessaire, de rediriger un peu notre conversation.
- Il se peut que vous trouviez les questions un peu répétitives au fil de l’entretien, je m’en excuse d’avance.
- Comme pour le premier entretien, nous allons enregistrer notre conversation. L’enregistrement nous permettra de retranscrire notre conversation afin d’analyser vos réponses. Il sera détruit à la fin de la recherche.
- Avez-vous une question avant que nous commencions?</td><td>Contextualiser le cadre et l’objectif de l’entretien (différencier l’objectif de recherche de cet entretien de la consultation qui a eu lieu).

Anticiper une conduite structurée de l’entretien et le temps imparti.

Répondre aux éventuelles questions complémentaires du patient.</td></tr>
<tr><th>THEMES</th><th>QUESTIONS & RELANCES</th><th>Intentions</th></tr>
<tr><td>ENTREE EN MATIERE</td><td></td><td></td></tr>
<tr><td>Motivation à participer et compréhension du contexte</td><td>Pourquoi avez-vous accepté de participer à cet entretien?
→ Qu’avez-vous pensé de cette proposition?
→ Quels sont les éléments présentés qui vous ont décidé à participer?
De manière générale, comment s’est passé cet entretien?
Annoncer que nous allons maintenant passer à des questions plus spécifiques.</td><td>Entamer la discussion.
Explorer les motivations initiales des patients.
Explorer la compréhension par le patient du contexte clinique et des objectifs du clinicien.</td></tr>
<tr><td>EXPERIENCE DE L’IM</td><td></td><td></td></tr>
<tr><td>Contexte</td><td>Est-ce que vous pourriez me décrire le contexte concret ou « la scène » de l’entretien (comme si vous en faisiez un tableau)?
→Qu’étiez-vous en train de faire quand la psychologue vous a proposé de discuter?
→ Moment de la prise en charge, endormi, réveillé, en soins?
→ Lieu, configuration des protagonistes, assis, couché?
Quel contexte aurait selon vous été idéal?
→ Autre clinicien, médecin des urgences?
→ Par téléphone? à la maison?
→ Après quelques jours?
→ Autre contexte que les urgences?</td><td>Vérifier la faisabilité des IM au Service des urgences.
Vérifier quelles modalités contextuelles/logistiques sont les plus adéquates.</td></tr>
</table>

Vécu général	**De façon générale, comment avez-vous vécu cet entretien?**<br>→ à l'aise? opportun? durée? intimité?	*Evaluer le sentiment de confort du patient dans le cadre de l'IM, en termes de contexte et/ou de contenu.*<br>➔ *Encourager un maximum de réponses spontanées à ce moment de l'entretien.*
Vécu du modèle: l'esprit motivationnel et les facteurs relationnels	**Que diriez-vous de l'attitude générale de la psychologue lors de cet entretien?**<br>→Empathique, attitude non-jugeante<br>**Dans quelle mesure vous vous êtes senti à l'aise avec cette personne?**<br>→ Confiance, compréhension, écoute	*Explorer la perception de l'attitude du soignant par le patient?*<br>*Evaluer l'adéquation entre l'esprit motivationnel et les attentes du patient.*
Vécu du modèle: prendre le temps	**Que diriez-vous de la qualité de l'entretien?**<br>→ Temps suffisant? exploration en profondeur de la situation individuelle?	*Vérifier si une IM unique dans le cadre des urgences permet de créer une relation significative.*
Vécu du modèle: contenu / schéma d'entretien et ses 3 étapes	**Si vous deviez résumer le contenu de l'entretien que vous avez eu à un ami, que lui diriez-vous?**<br>→ Sujets spécifiques abordés, Début? Fin?<br>→ Différentes étapes dans l'entretien?<br>→ But spécifique de l'entretien?	*Evaluer la perception d'une intervention en plusieurs phases (la dynamique de l'entretien et l'évolution de la consultation).*<br>*Evaluer la perception de la finalité de l'intervention.*
Vécu du modèle: Attribution causale	**La discussion vous a-t-elle amené à faire un lien entre votre consommation d'alcool et votre admission aux urgences?**<br>→ Comment le sujet a-t-il été abordé?<br>→ Par qui et à quel moment de l'entretien?	*Evaluer la perception de l'attribution causale.*
Vécu du modèle: Informations par rapport aux risques liés à l'alcool	**Lors de cet entretien, quelles informations avez-vous reçues au sujet des risques liés à l'alcool?**<br>→ Qu'en pensez-vous?<br>→ Qu'allez-vous en faire?<br>→ Comment vous êtes-vous senti à ce moment de l'entretien?	*Evaluer l'application et la pertinence de donner des informations.*
Vécu du modèle: conseils	**Dans quelle mesure l'intervenante vous a-t-elle donné un ou des conseils par rapport à votre consommation d'alcool?**<br>→ Si oui, lesquels? / qu'en pensez-vous? / comment vous êtes-vous senti?<br>→ Si non, quels types de conseils auriez-vous jugé utiles?	*Evaluer l'application et la pertinence de donner des conseils au patient.*
Vécu du modèle: le discours changement/planification	**Dans quelle mesure avez-vous évoqué des choses que vous aimeriez mettre en place dans votre vie?**<br>→ Dépassement de l'exploration de la situation présente.<br>→ De quels changements avez-vous parlés?	*Vérifier la pertinence de dépasser la situation actuelle dans le cadre de l'IM et de se projeter dans le temps.*<br>*Explorer le sentiment de confort du patient dans l'exploration du changement.*

	**Comment avez-vous vécu cette partie de la discussion?**<br>→ Résistances? stress? sentiment d'intrusion?	
Vécu du modèle: influence de l'entourage	**Dans quelle mesure avez-vous parlé de vos proches (amis, famille) pendant l'entretien?**<br>→ Soutien/obstacle dans la réalisation du changement?<br>→ Qu'en pensez-vous?<br>**Comment avez-vous vécu cette partie de la discussion?**	*Evaluer l'application et la pertinence d'impliquer l'entourage pendant l'entretien (sans qu'ils soient présents).*
Impact de l'IM	**Globalement, quelles sont les choses dont vous avez discutées pendant cet entretien qui ont été/seront utiles pour vous ou que vous avez appréciées?**<br>→ Et celles inutiles, pas appréciées?<br>→ Est-ce que qqch vous a amené à voir votre situation différemment?<br>**Comment pensez-vous que vous repenserez à cet entretien demain?**	*Evaluer l'impact immédiat de l'IM.*<br>*Mettre en évidence les points forts et les points faibles en termes de sentiment d'utilité pour le patient de l'IM.*
SUITE DE LA PRISE EN CHARGE		
Modalités de suivi	**Qu'avez-vous convenu comme suite?**<br>→ Qu'en pensez-vous?<br>→ Auriez-vous d'autres suggestions/envies/attentes?<br>**Que pensez-vous du fait de recevoir un compte-rendu de l'entretien?**	*Explorer les modalités de suivi les plus adaptées.*<br>*Compléter/adapter les modalités de suivi envisagées.*<br>*Vérifier l'intérêt des patients à poursuivre la discussion et la possibilité d'initier une projection dans le temps.*
SUGGESTIONS DE CHANGEMENT	Contenu:<br>**Qu'auriez-vous souhaité qu'il se passe idéalement dans cet entretien?**<br>→ Auriez-vous souhaité bénéficier d'une évaluation quantitative de votre consommation et d'un retour?	*Évaluer les attentes du patient en termes de prise en charge, en termes de contenu.*<br>*Explorer les éléments contextuels et de contenu inadaptés aux attentes/à l'état/aux capacités du patient.*
QUESTION DE CLOTURE	**Y a-t-il autre chose que vous souhaiteriez partager concernant l'entretien avant que nous terminions cet entretien?**	*Faire ressortir les éventuels éléments importants non abordés directement par l'enquêteur.*<br>*Terminer l'entretien de façon douce en donnant la parole à l'interviewé.*
