# Supplementary material for: Developing a brief motivational intervention for young adults admitted with alcohol intoxication in the emergency department – Results from an iterative qualitative design
Source: PLoS One. 2021 Feb 8;16(2):e0246652. doi: 10.1371/journal.pone.0246652 (PMC7869998; doi:10.1371/journal.pone.0246652)
Supplement: S1 Table — (PDF) [file pone.0246652.s002.pdf]

S1 Table

Original quotes in French

Quotes from Round 1	
Q1.	Ca peut être difficile de reprendre le fil, de savoir où on en était, de qu’est ce que j’avais en tête que je voulais dire, qu’est-ce que le patient était en train de dire.
Q2.	L’impression en fait qu’en vous accordant du temps à vous, on perd du temps en pouvant sortir plus vite.
Q3.	Je vois pas dans quel autre contexte il y aurait pu avoir une discussion pareille.
Q4.	C’est pas… enfin c’est vraiment pas un truc anodin quoi et je pense il faudrait être un peu je sais pas, simplet [rires], stupide pour ne pas, enfin pas considérer ça comme, […] c’est pas rien quoi, c’est, je suis aux urgences, c’est pas pour rien et il y a un problème, voilà, ça pousse vraiment à réfléchir, à opérer un changement.
Q5.	Disons que moi j’avais ça en tête, après c’est en fonction de ce que la personne amène qu’on fait des aller et retour, mais sinon ça me paraît assez cohérent, une construction logique.
Q6.	J’ai pas eu l’impression de pouvoir aller très loin dans la discussion autour de l’alcool, de ce qui faisait qu’elle était ici aux urgences ce jour-là.

Q7.	Quand je lui ai demandé comment elle voyait ses consommations à l'avenir, elle arrivait pas forcément à me répondre, et puis heu, bon elle disait que ça allait aller de mieux en mieux, mais du coup dans la planification y avait pas vraiment un objectif concret, voilà, mais. J'ai quand-même demandé quelle était l'importance pour cette personne, à quel point elle se sentait confiante, donc voilà je faisais, ça a été mais c'est vrai que y avait pas forcément quelque chose de concret non plus.
Q8.	A mon avis ça pourrait être très dissuasif pour certaines personnes. Quand on voit la fourchette 20 à 60 minutes, ça fait quand-même peur !
Q9.	Je suis sûre qu'il y a des gens en fait qui ont envie de faire votre truc mais qui se disent que non, ils vont perdre leur temps, que ça va leur faire perdre encore plus de temps...
Q10.	C'était pas comment dire, intrusif ou, elle cherchait pas quelque chose, elle cherchait pas à obtenir quelque chose et, bein voilà c'était une relation, […] je pouvais vraiment parler normalement.
Q11.	Elle, elle juge pas, elle prend juste les informations.
Q12.	Ouais, moi ça m'aurait intéressé qu'elle me donne son point de vue par rapport à ce que j'ai dit. Parce qu'au final elle m'a juste écouté, elle m'a posé des questions, mais m'a donné aucun avis en fait.
Q13.	Ça pourrait être utile, oui […].ben d'indiquer, heu, de savoir où on se trouve quand-même réellement parce que, je pense qu'il y a beaucoup de personnes qui pensent qu'ils boivent pas beaucoup pis qu'au final c'est déjà des bons consommateurs.

Q14.	– Je me suis sentie à vouloir lui donner en fait plus je, heu, peut-être j'aurais pu me le permettre hein, mais j'aurais eu envie de lui donner plus d'informations en fait.<br>– Sur quoi ?<br>– Sur le fait que, quand elle dit que pour elle une consommation tout-à-fait normale et non problématique c'est finalement quelque chose d'assez élevé ; heu j'aurais eu envie de lui donner, de lui faire ce retour-là, pour qu'elle se situe par rapport à une certaine norme en fait parce que sans ça, bein peut-être que j'aurais dû me le permettre hein, mais sans ça c'est presque un peu si j'entendais ce qu'elle consommait mais que je normalisais aussi ou...[…] mais mon questionnement c'était est-ce que j'ose le faire tout en restant dans une approche motivationnelle.
Q15.	Je pense qu'il faut faire attention dans le cas où, où vous l'enverrez en fait. Parce qu'il y a des gens, moi par exemple, moi si vous me l'enverrez je vais me sentir plus blessée, genre en gros blessée dans le sens où vous me voyez comme une alcoolique, et ça moi je vais mal le prendre, ça va me blesser parce que je sais que je suis pas comme ça et ça m'énerve.
Q16.	Ça fait du bien de voir, enfin de pouvoir penser à plus loin, après ça, parce que ben je me serais jamais imaginé ici donc heu, j'aurais pas non plus imaginé devoir penser à un changement parce que je suis ici, quoi

Q17.	Heu ça fait du bien de, on s'en rend peut-être pas compte heu avant mais ça fait, ça fait du bien de heu de pouvoir parler, de de ce qui s'est passé ouais c'est toujours bien de parler quoi de… Garder les choses pour soi c'est pas bon et pis si on peut parler à une personne extérieure c'est toujours bien.
Q18.	Ça fait réfléchir quoi, toujours et, ben c'est pas anodin, c'est quand-même une drogue, pis, même si c'est accepté par la société je veux dire, quand-même réussir à trouver sa juste mesure, enfin, voilà, réussir à adapter sa consommation […], pas avoir le verre de trop.
Q19.	J'ai essayé de mettre en lien, bein quand-même c'est quelqu'un qui a fait une chute dans les escaliers, heu alcoolisée, en fin de soirée, pis au départ elle disait c'est juste malheureux, ça n'a rien à voir avec l'alcool… Alors en fin d'entretien elle a quand-même dit qu'effectivement si elle avait pas bu elle serait pas tombée. Je me suis dit en tous cas que peut-être à quoi ça avait servi c'est qu'elle le verbalise… Et qu'elle s'entende dire « effectivement peut-être que si j'avais pas bu je serais pas tombée ».
Q20.	Évidemment je réfléchis à ce que j'aurais pu faire différemment, à ce que m'a dit la patiente et puis au fait que j'ai pas l'impression qu'elle a beaucoup avancé pendant cette intervention.
Quotes from Round 2	
Q21.	Euh alors j'aurais préféré le faire maintenant, vu en fait comment j'suis maintenant, enfin je regrette d'être sortie, enfin d'avoir pris certaines choses avant, donc j'préférerais l'faire maintenant que attendre un jour et qu'j'sois plus reposée.

Q22.	Bah je pense qu'il est pas possible de tout faire en une intervention aux urgences, en fait, parce que la personne est toujours dans un état de, voilà, de choc, il y a l'alcool certainement encore en jeu, y a l'émotion, […] je pense que de, de reprendre les choses plus tard, quand la personne a pu réfléchir, voilà, je pense que c'est, que ce serait d'autant, ce serait pas mal, efficace.
Q23.	Elle m'a laissé m'exprimer […] je me suis pas senti oppressé ni… enfin, comment dire, restreint par le temps ou quoi que ce soit.
Q24.	Je trouve que c'est bien […], ça montre aussi en fait un investissement par rapport à, ben en fait aux deux personnes.
Q25.	C'est pas comme si c'était des questions écrites, ça veut dire si je dis quelque chose elle va me poser une question par rapport à ça.
Q26.	Ben je savais déjà qu'il y avait pleins de risques [rires], donc j'ai pas trop, j'ai pas trop pensé, je savais déjà.
Q27.	J'lui ai d'mandé un p'tit peu si elle connaissait les effets et pi euh, et pi, et pi on a parlé un p'tit peu d'ça. J'suis pas allée dans l'détail parce que j'ai pas eu l'impression qu'elle avait besoin de plus que c'qu'elle avait.
Q28.	Une fois que je lui ai demandé ce qu'il pensait du fait de se retrouver là aux urgences etc. voilà j'ai pensé que c'était assez bien placé pour faire ça et pis il a été preneur donc je lui ai, oui. […] Je lui ai demandé s'il était d'accord justement qu'on parle des risques liés à la consommation d'alcool et quand il m'a dit oui, j'ai commencé par lui demander ce que lui il savait. […]. Et du coup lui il t'a spontanément donné enfin dit

	un peu les risques qu'il connaissait par rapport à l'alcool […] et du coup je lui ai demandé s'il était d'accord que je complète ce que lui avait, tout en validant bien-sûr ce que lui avait dit.
Q29.	J'ai l'impression que cet entretien lui a fait aussi beaucoup de bien. Enfin comme il a beaucoup parlé, je crois qu'il avait vraiment besoin de déposer, et puis je pense que ça lui a fait du bien qu'il y ait quelqu'un qui, qui puisse l'écouter aussi.
Q30.	C'qui m'a un peu aidé c'est, justement à comprendre pourquoi j'suis là et, pourquoi j'ai fait ça.
Q31.	Et c-ça m'a fait comprendre qu'il faut vraiment faire attention à c'qu'on boit. […] il faut pas non plus trop boire, trop vite, il faut surtout bien s'hydrater, bien manger.
Q32.	Hum, ben, de toutes façons j'étais déjà dégoûtée moi-même, par moi-même, que j'allais pas sortir, donc c'est pas l'entretien qui a influencé.
