# Supplementary material for: Developing a brief motivational intervention for young adults admitted with alcohol intoxication in the emergency department – Results from an iterative qualitative design
Source: PLoS One. 2021 Feb 8;16(2):e0246652. doi: 10.1371/journal.pone.0246652 (PMC7869998; doi:10.1371/journal.pone.0246652)
Supplement: S2 Table — (PDF) [file pone.0246652.s003.pdf]

S2 Table

Ideas generated by experts and experts' scoring of these ideas using the Nominal Group Technique

Topic 1 – “Now that you have the intervention model in mind, what would you add and/or modify?”

Ideas generated	Experts’ score									N votes	Total score
	Exp. #1	Exp. #2	Exp. #3	Exp. #4	Exp. #5	Exp. #6	Exp. #7	Exp. #8	Exp. #9		
Add / focus on follow-up contact.		3	2		1	1	3	5		6	15
Provide a simple, structured, replicable model.	5	5	1			5	4			5	20
Give advice/direction to patients (with permission).				4	5	2		2		4	13
Work on social support (discussion on where clients can get social support).		1	4		3		1			4	9
Give patients a tablet (with electronic intervention contents): discussion, tablet, discussion.				5		4		4		3	13
Focus on precipitating event; Work on causal attribution of ER admission to alcohol intoxication.	3		5						3	3	11
Provide something patients can take with them (e.g. written change plan, letter to themselves, recording of the session, other?).	2							1	2	3	5
Give age-matched normative feedback.					4			3	2	3	9
Restrict age group. Narrow to 18-24 years old.	1						5			2	6
Make connections (target not necessarily to change or fix problems, but to engage people and make connection).		2				3				2	5
Getting someone else involved (e.g. peer, significant other).					3				1	2	4
Give information about what they are in the ER for (information about alcohol intoxication).				1	2					2	3
Give advice about avoiding coming back to ER.					5					1	5
Work on agenda setting.									4	1	4
Consider computer-based intervention for less severe patients.								4		1	4
Discuss issue of self-control and relapse within the planning phase (e.g. previous attempts, failures, success, self-efficacy).		4								1	4
Think about qualities of clinicians: anticipate therapist effects.	4									1	4
Encourage to look to the future.				3						1	3
Approach the group, not just the individual.			3							1	3
Work on peer influence.							2			1	2
Work on evocation and planning together (instead of two different steps in the current model).				2						1	2
Work on identity development.										0	0
Think about how topic is broached.										0	0
Meaning, values, life fulfilment vs alcohol.										0	0
Target different goals depending on severity.										0	0
Provide ideas/options about what they might do (with permission).										0	0
Do not distinguish between drinkers (same intervention model for all).										0	0
Think about the setting: Separate room for intervention? Take the patient out of the ER? Other context?										0	0
Change talk: train clinicians to discriminate and work with genuine change talk.										0	0

Topic 2 – “How would you provide information?”

Ideas generated	Experts’ score									Total score	N votes
	Exp. #1	Exp. #2	Exp. #3	Exp. #4	Exp. #5	Exp. #6	Exp. #7	Exp. #8	Exp. #9		
Highly individualized information, “letter” given to clients at the end of the intervention.	4			3			3	5		15	4
No leaflet. They are young adults, use smartphone to send them messages (tailored on what was discussed).	3	2			2	5				12	4
Computer interactive Elicit-Provide-Elicit exercise, tailored to outcomes.	5		3	2				1		11	4
Sending information from tablet (used during BMI) to client’s smartphone.	3					4	5			12	3
What information: give tailored advices, with options, and specific not generic information.	4		4		3					11	3
Video of a peer (age/gender-matched): appealing, positive story on something they can do since they are not intoxicated (vs. negative story e.g. car crash).				4	5				2	11	3
Simple, replicable, standardized information.	5	3				2				10	3
Multiply contacts and media (SMS, email, telephone).	3		2						5	10	3
Provide information on tablet, with graphs, visually appealing.		4					1		1	6	3
Computerized: friendlier. Let client decide what information is most useful.			1	1					4	6	3
Information about intoxication: specific to client BAC; normative feedback about intoxication.				5	4					9	2
Personalized “exercises” on options and strategies, in a booklet, then discussed. Make it personal.		1				3				4	2
To whom: give some information to everyone, with permission.					1				2	3	2
Feedback on what they said (or record of the session) – People sometimes forget what they talked about during BMI.			5							5	1
Information on risk levels, normative feedback, sources of help.		5								5	1
Referral information: options, suggestions (not only on treatment).							4			4	1
Personalized pamphlet: only information that are relevant to the patient. Graphical when possible.	4									4	1
Include information on process of change, psychological constructs, feedback on intra-individual changes.								4		4	1
Share evidence-based information on alcohol use and consequences (ethical obligation).									3	3	1
When: after engagement.								3		3	1
Improve on what’s already good.	2									2	1
Minimal information to everyone. Additional information depending on what people are interested in / need.								2		2	1
Audio recording (client talking to themselves).							2			2	1
Information about referral doesn’t work. If referral is needed, make the appointment.						1				1	1
Provide printed information.										0	0
Provide written information (well-presented leaflet), can be provided online.										0	0
Involve other people if they are available, group discussion.										0	0
Information/advice about how to cope with temptation/relapse – after commitment to change .										0	0

Topic 3 – “What would you add/modify in the intervention booster model?”

Ideas generated	Experts’ score									Total score	N votes
	Exp. #1	Exp. #2	Exp. #3	Exp. #4	Exp. #5	Exp. #6	Exp. #7	Exp. #8	Exp. #9		
Electronic delivery of the booster letter (send SMS instead of letter).		4	4		4		3	3	4	22	6
Ensure some follow-up. Ensure health care contact soon after ER (in primary care) a few days later (=initial intervention for those not remembering what happened in the ER).		5	2		5	1		5		18	5
Keep it simple/feasible.	5		1	4		5				15	4
Include fresh data, individualized.				5			2	4	3	14	4
Less is better! Limit follow ups since some patients do not want them.						2	1	2	5	10	4
Smartphone app.	1	3			4					8	3
Make it personalized.	3			3					2	8	3
Ask permission; send tailored SMS each day for a given period, give permission to turn it off.			3				4			7	2
Consider transferring referral to primary care (away from specialized care).		1	5							6	2
Make it memorable.	4			2						6	2
Involve other people: ask permission to send SMS to others.		2							2	4	2
Multi modal.	2			1						3	2
Ask client what would be useful for them to receive in a text.							5			5	1
Use new technologies: email pdf, not letters; texts, not phone calls.						4				4	1
Online. Use text to remind appointments, collect data…						3				3	1
Ask them to do a video of themselves to remind themselves of something they had “decided”.									1	1	1
Be proactive.								1		1	1
Include both letter and phone calls.										0	0
Provide the option to use an app and/or speak with someone on the phone.										0	0
